# DiScO: novel rapid systems mapping to inform digital transformation of health systems

**DOI:** 10.3389/fpubh.2024.1441328

**Published:** 2024-10-25

**Authors:** Tarun Reddy Katapally, Nadine Elsahli, Jasmin Bhawra

**Affiliations:** ^1^DEPtH Lab, School of Health Studies, Faculty of Health Sciences, Western University, London, ON, Canada; ^2^Department of Epidemiology and Biostatistics, Schulich School of Medicine and Dentistry, Western University, London, ON, Canada; ^3^Lawson Health Research Institute, London, ON, Canada; ^4^CHANGE Research Lab, School of Occupational and Public Health, Toronto Metropolitan University, Toronto, ON, Canada

**Keywords:** digital citizen science, decentralization of technology, democratization of data, digital transformation of health systems, systems integration, systems mapping, systems thinking

## Abstract

**Background:**

Global health systems are confronting challenges that intersect climate change with evolving communicable and non-communicable public health risks. Addressing these challenges requires systems integration via citizen big data that exist outside health systems. However, systems integration across jurisdictions is a complex challenge that requires stakeholder input. This study’s purpose was to conduct rapid systems mapping with international health system stakeholders to inform the development and implementation of a global digital citizen science observatory (DiScO), which aims to catalyze digital transformation of health systems across jurisdictions.

**Methods:**

A rapid qualitative systems mapping study was conducted during the International Society for Behavioral Nutrition and Physical Activity Annual Global Summit in Uppsala, Sweden, in June 2023. The choice of the venue and approach was informed by three key criteria: (1) Established evidence linking physical activity and nutrition with non-communicable diseases; (2) Concrete existing methods of obtaining citizen big data by physical activity and nutrition researchers; (3) Precedence of physical activity and nutrition researchers conducting citizen science as well behavioral/clinical big data collection. The design of this study was an innovative pre-post systems map development, which consisted of (1) real-time rapid systems mapping (pre/initial map) by engaging with international stakeholders and (2) adjustment of the real-time systems map (post/final map) after analyzing stakeholder discussion data.

**Results:**

Rapid systems mapping resulted in a complex network that included key themes to successfully develop and implement DiScO: priorities, opportunities, risks, challenges, partnerships, and resources. Additionally, a new theme emerged organically through stakeholder group discussions – mitigation strategies. The adapted rapid systems map (i.e., after data analyses) depicts 23 key nodes of intervention across the seven key themes.

**Conclusion:**

Rapid systems mapping at international symposia is a novel methodological approach to capture stakeholder input, particularly to understand complexity across international jurisdictions – an approach that can be replicated across disciplines and sectors to inform digital transformation of health systems. The development and implementation of DiScO, a platform for decentralization and democratization of technology, will take into consideration all the key nodes of intervention identified in the rapid systems map to promote digital health for equity across global jurisdictions.

## 1 Introduction

Globally, health systems are confronting challenges that intersect climate change with evolving communicable and non-communicable public health risks (1–4). The escalating frequency and severity of climate-related weather events, such as heatwaves, cyclones, and droughts, are amplifying pre-existing health inequities. In turn, this polycrisis – defined as multiple global crises interacting and overlapping to exacerbate the effect of one another (2, 5) – is increasing the burden on existing healthcare infrastructure, while increasing consumption of resources (1, 2). The current polycrisis calls for comprehensive strategies to adapt public health systems through the coordination of decision-making with systems outside of healthcare (6).

In particular, health systems worldwide are struggling to meet the evolving needs of the public, and continue to fall short in delivering timely and effective healthcare (7–10). Moreover, these systems tend to overlook preventive practices (11), perpetuating reliance on healthcare services (12). These inefficiencies stem from limitations in traditional healthcare systems, which lack systems thinking approaches (10, 13). Specifically, in healthcare, systems thinking involves understanding how several components within a system interact to influence behaviors or outcomes (14). For instance, socioeconomic status, access to care, and public health policies can have impacts on individual and population health outcomes (15). According to the World Health Organization (WHO), these inefficiencies necessitate a shift toward multisectoral policies through integrated systems (16). The application of systems thinking in recognizing the interrelatedness of various factors that impact individual and community health can enable systems integration (17). This approach helps to address complex problems that go beyond individual disciplines and sectoral boundaries by considering relationships between downstream factors and broader social forces that shape health outcomes at both individual and population levels (18). For instance, social determinants of health such as education, socioeconomic status, and physical environment have been shown to significantly influence health behaviors, access to healthcare services, and overall health outcomes (19, 20). However, to truly trace connections between community-specific factors and pinpoint crucial health system intervention points (21), consistent big data from systems outside healthcare are necessary – a critical gap that exists across global health systems (22, 23).

Digital citizen science, a social innovation approach (6), which involves active contribution of citizens in conceptualization of health systems interventions can transform ethical big data collection via citizen-owned ubiquitous internet-connected devices (e.g., smartphones) (24, 25). This approach has the potential to support digital transformation of health systems by integrating big data across systems, i.e., big data obtained from citizens to capture information about both health systems as well as systems outside of health (e.g., food, environment, education) (6, 22, 26, 27) in the form of text, image, video, or audio. Ethical big data collection via citizen-owned ubiquitous internet-connected devices can ultimately facilitate real-time health-related information, while facilitating remote engagement between citizens and decision-makers (22). However, this approach also raises concerns regarding data privacy, security, and regulatory compliance (22). Cross-jurisdictional data legislation variations also hinder the exchange of data between, and even within international jurisdictions (i.e., countries and specific states and provinces within them which abide by jurisdictional legislations specific to them) (28–30). Not to mention there is a need for ethical incentivization of citizens to obtain big data (24, 31, 32); this is currently a significant barrier to digital transformation of health systems (33).

To address challenges in citizen-driven big data and evidence-based decision-making within and across jurisdictions, the Digital Epidemiology and Population Health Laboratory (DEPtH Lab) is developing a Digital Citizen Science Observatory (DiScO) (5). This platform, which is funded by the Canada Foundation for Innovation (34), aims to facilitate digital transformation of health systems by ethically utilizing citizen-driven big data to predict and prevent public health crises across jurisdictions. DiScO’s approach involves scaling up/down, replicating, and re-purposing existing digital technology (22, 35) to prioritize and promote open science (36), and the decentralization as well as democratization of technology and big data (5, 6, 20). However, development and implementation of DiScO on a global scale requires comprehensive global stakeholder input to understand and account for the complexity of implementing digital health infrastructure across international jurisdictions (37) – a challenging and time-consuming operation, which can delay digital transformation needed to address existing evolving public health crises.

One approach for obtaining rapid stakeholder input, particularly to capture systems thinking, is “systems mapping” (38). Systems mapping serves as a practical qualitative tool to operationalize systems thinking by organizing, visualizing, and clarifying complex discussion areas (39, 40). Creation of a systems map enables discussions among intersectoral stakeholder groups about a topic or problem, and importantly depicts connections between factors across multiple levels and systems to visually illustrate the patterns and directions of cause and influence on certain outcomes (41). Evidence to date shows that systems mapping exercises are a valuable tool in capturing the complexity of problems and incorporating diverse stakeholder perspectives (42, 43). Additionally, it has been used to shape implementation of programs and policies across disciplines and sectors, including initiatives to address non-communicable diseases (NCDs), HIV drug resistance, among others (42, 44, 45). These exercises not only help stakeholders understand the multifaceted nature of challenges, but also identify leverage points for intervention, prioritize actions, and facilitate collaboration across disciplines (46, 47). In addition to shaping program and policy development, systems mapping can facilitate transparency in decision-making processes by making the rationale behind choices visible to all stakeholders (42). However, to our knowledge, no systems mapping has been conducted thus far to engage global stakeholders to develop and implement digital infrastructure across international jurisdictions to monitor, mitigate, and manage complex public crises. To address this gap, the aim of this study is to employ a systems thinking approach to engage decision-makers and researchers across international jurisdictions to develop an evidence-based rapid systems map for the development and implementation of DiScO.

## 2 Methods

### 2.1 Study design

The design of this study was an innovative pre-post systems map development, which consisted of (1) real-time rapid systems mapping (pre/initial map) by engaging with international stakeholders and (2) adjustment of real-time systems map (post/final map) after analyzing stakeholder discussion data. The study was conducted at the International Society for Behavioral Nutrition and Physical Activity Annual Global Meeting in Uppsala, Sweden, in June 2023 (48). The choice of the venue and approach was informed by three key criteria: (1) Established evidence linking physical activity and nutrition with both communicable and non-communicable diseases (49–52); (2) Concrete existing methods of obtaining citizen big data by physical activity and nutrition researchers by working across systems (health, food, education etc.) (53, 54); (3) Precedence of involvement of physical activity and nutrition researchers in both citizen science as well behavioral and clinical big data collection with citizen-owned internet-connected digital devices. The summit has a record of including international experts, both researchers and decision-makers/policymakers (i.e., stakeholders) in its agenda, which come from more than 150 academic and medical institutions, as well as approximately 40 government agencies, industry, and professional organizations (55). In essence, the rationale for the stakeholder selection and venue was based on application of findings across disciplines, ability to obtain expert evidence from across international jurisdictions, and replicability of rapid systems mapping at international events.

### 2.2 Study setup

**Figure 1:**
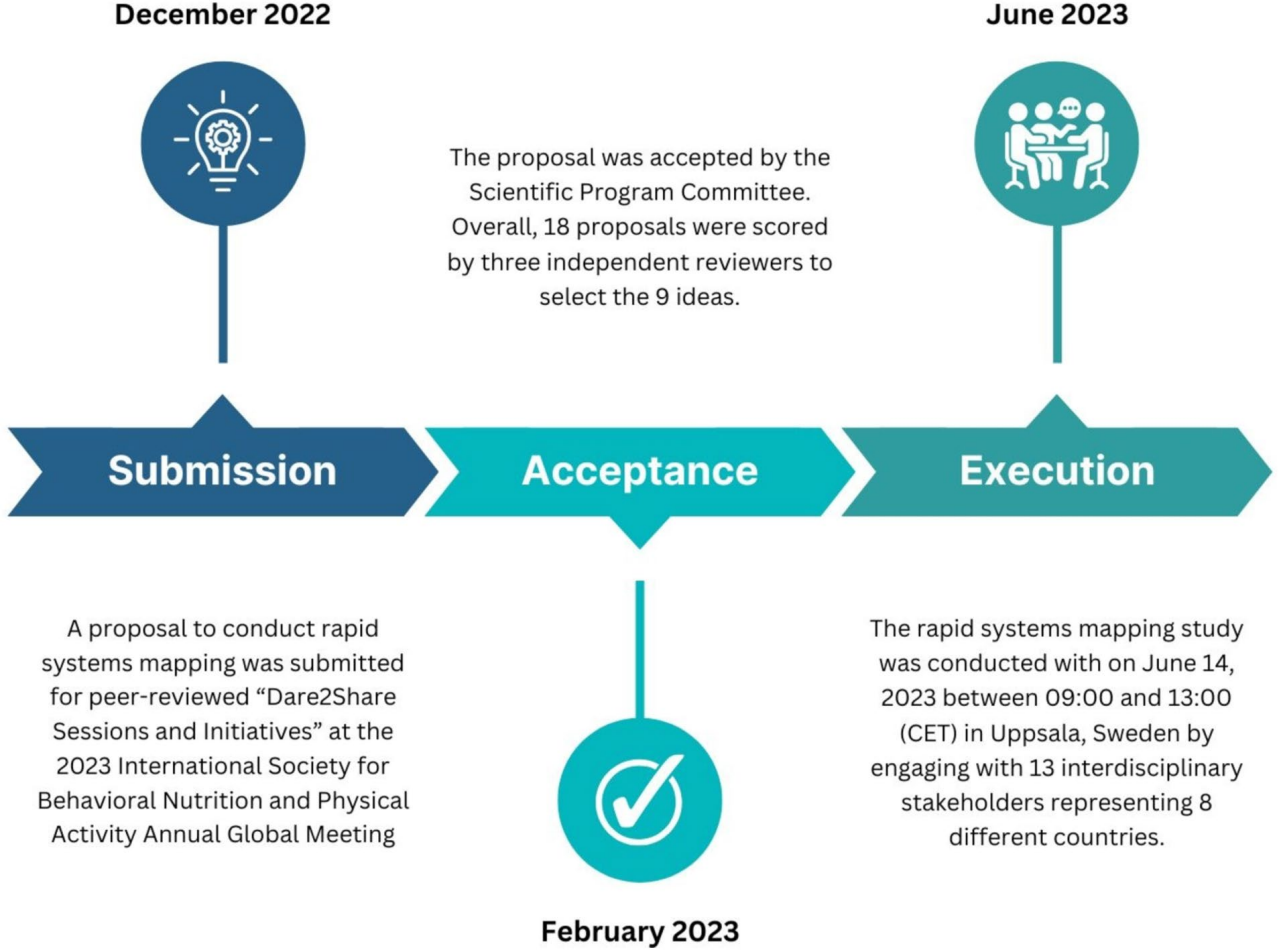
Timeline of the DiScO systems mapping study.

A timeline of the study setup is shown in Figure 1. In December 2022, a DEPtH Lab proposal to conduct rapid systems mapping was submitted for peer-reviewed “Dare2Share Sessions and Initiatives” at the 2023 International Society for Behavioral Nutrition and Physical Activity Annual Global Meeting (56). According to the criteria, “Dare2Share sessions and initiatives need to be interactive, experiential, creative and innovative” (57). The criteria clearly indicated that only those proposals that were “out-of-the-box ideas” would be considered. The overall goal of the innovative sessions was to foster meaningful connections in the form of global collaborations, and to facilitate conversations that would drive innovation (56).

The DEPtH Lab proposal articulated the overall objectives of DiScO: (1) Expand a digital citizen science platform across international jurisdictions to create an ethical big data repository; (2) Utilize citizen science approaches to drive human-centered artificial intelligence to analyze and visualize big data in near real-time; (3) Inform behavioral and policy interventions across jurisdictions using big data. The proposal also articulated the Dare2Share session goal of conducting a rapid systems mapping study to create a global digital ecosystem by: (1) Identifying gaps and needs; (2) Discussing data sharing; (3) Addressing data ownership, privacy, and security; (4) Identifying available resources; and (5) Identifying potential partners across low-, middle-, and high-income countries.

The proposal was accepted by the Scientific Program Committee in February 2023. Overall, 18 proposals were scored by three independent reviewers to select the 9 ideas. Thereafter, the rapid systems mapping session was promoted via social media and directly to the Global Meeting attendees by the organizers. The Dare2Share Sessions were open to all Global Meeting attendees free of charge to encourage equitable participation from both global south and north stakeholders.

### 2.3 Rapid systems mapping: setting, recruitment, and data collection

A rapid qualitative systems mapping study was conducted with on June 14, 2023 between 09:00 and 13:00 (CET) in Uppsala, Sweden by engaging with 13 interdisciplinary stakeholders representing 8 different countries, including Australia, Canada, Estonia, Germany, India, Taiwan, France, and the United States. An open selection process, i.e., a self-selection process and free invitation to the session was utilized, allowing any interested stakeholders attending the summit to participate. The stakeholder roles varied from academic scientists and trainees to health system decision-makers and knowledge users. Participation in the systems mapping exercise was voluntary, and stakeholders were free to leave the session at any time without consequence. Consent to audio and video record the session was obtained from all stakeholders prior to the session, and steps were taken to protect their privacy during the recording of the session. No personally identifiable data was collected, and comments were kept anonymous.

The rapid systems mapping consisted of three broad phases in the following order: (1) Presentation of DiScO by the 3 moderators; (2) Two independent un-moderated group discussions; (3) One moderated overall discussion. Moderator 1 introduced DiScO (Figure 1) to the stakeholder audience and described its overall goal, existing digital infrastructure, development scope, potential benefits, and ultimate implementation and evaluation plans. The complete description and methodological approach of DiScO is available online: “It’s late, but not too late to transform health systems: A global digital citizen science observatory for local solutions to global problems” (5). In brief, Moderator 1, presented the approach to building DiScO, which is to scale-up/down, replicate or re-purpose existing digital health infrastructure, as relevant. This approach prioritizes and promotes open science (36) and rapid response structures irrespective of location of development and implementation. Following this replicability-focused approach, the DEPtH Lab is currently engaging in the first step of the development process. This step involves leveraging existing digital health infrastructure, which includes a digital platform which serves as a public health advisor. Moreover, the platform has been implemented via a novel progressive web application (PWA) that can be modified to provide jurisdiction-specific public health advice (5, 22). The PWA is linked to a digital health dashboard via cloud-based data servers. The development process resulted in a replicable and scalable jurisdiction-specific digital health platform that was tested to confirm not only the real-time linkage between the PWA and the digital health dashboard, but also the functionality of the PWA in its ability to provide citizens with real-time public health advice. Simultaneously, it relays aggregated and anonymized big data to the digital health dashboard to enable jurisdictional decision-making, i.e., operationalization of digital citizen science while providing value to both citizens and decision-makers. This approach to citizen science for the development of digital health dashboards enables new opportunities, such as scaling the dashboards up to implement DiScO across jurisdictions to address public health crises from a systems perspective.

**Figure 2:**
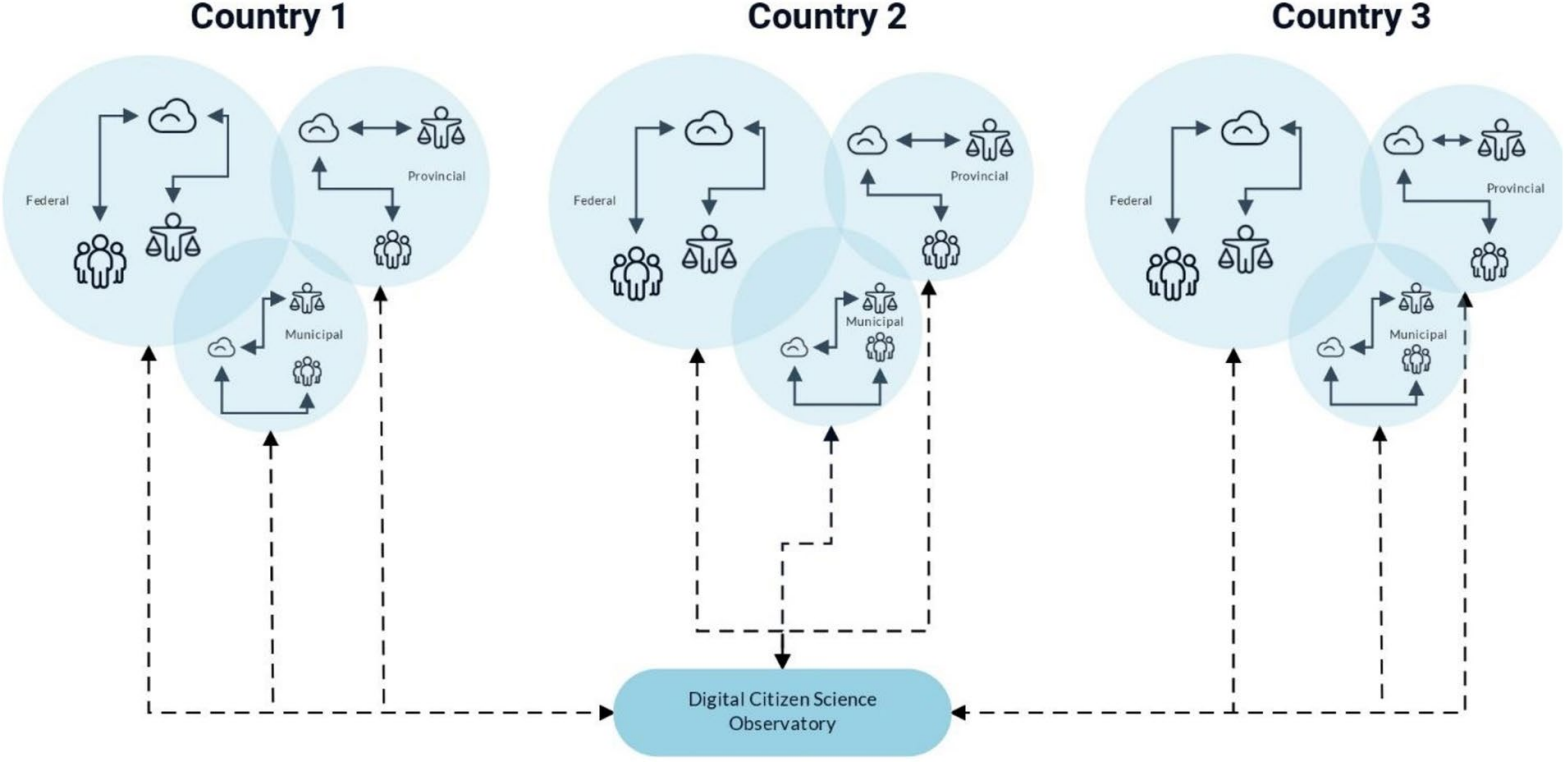
DiScO showing data flow across and within jurisdictions.

Figure 2 enumerates the DiScO data flow, with the solid arrows depicting big data exchange within jurisdictions highlighting decentralized and jurisdiction-specific cloud-based collection and storage of big data. The standardized and scalable digital infrastructure is shown by dotted arrows going from the central cloud-based repository to individual jurisdictions (platform updates, bug fixes, new features etc.). The dotted arrows going from individual jurisdictions to the central repository portray data sovereignty of jurisdictions, where only completely de-identified, and irreversibly anonymized data can be centrally shared to facilitate global solutions, while abiding by individual jurisdictional data regulations.

Moderator 2 provided the stakeholder audience with real-world application of DiScO by describing how the observatory is not only developed to address needs of the citizens, but also to meet decision-maker goals in addressing citizen needs (37). Moderator 2 enumerated that the same digital health infrastructure could be modified to address jurisdictional-specific issues ranging from climate change to infectious diseases (22, 24) and non-communicable diseases (35) to systemic issues such as food security (26) – an approach that currently lies beyond the scope of health systems. Finally, Moderator 2 detailed the overall benefits of DiScO: (1) Social and Societal Benefits: (a) Citizen and Community Empowerment; (b) Big Data-Enabled Health Promotion; (c) Misinformation Management. (2) Research Benefits: (a) Rapid Evaluation of Population Health Interventions; (b) Real-Time Behavioral Interventions; (c) Methodological and Analytical Innovation; and (3) Advocacy and Policy Benefits: (a) Real-Time Knowledge Translation; (b) Rapid Decision Making; (C) Internet Equity and Data Sovereignty (5).

**Figure 3:**
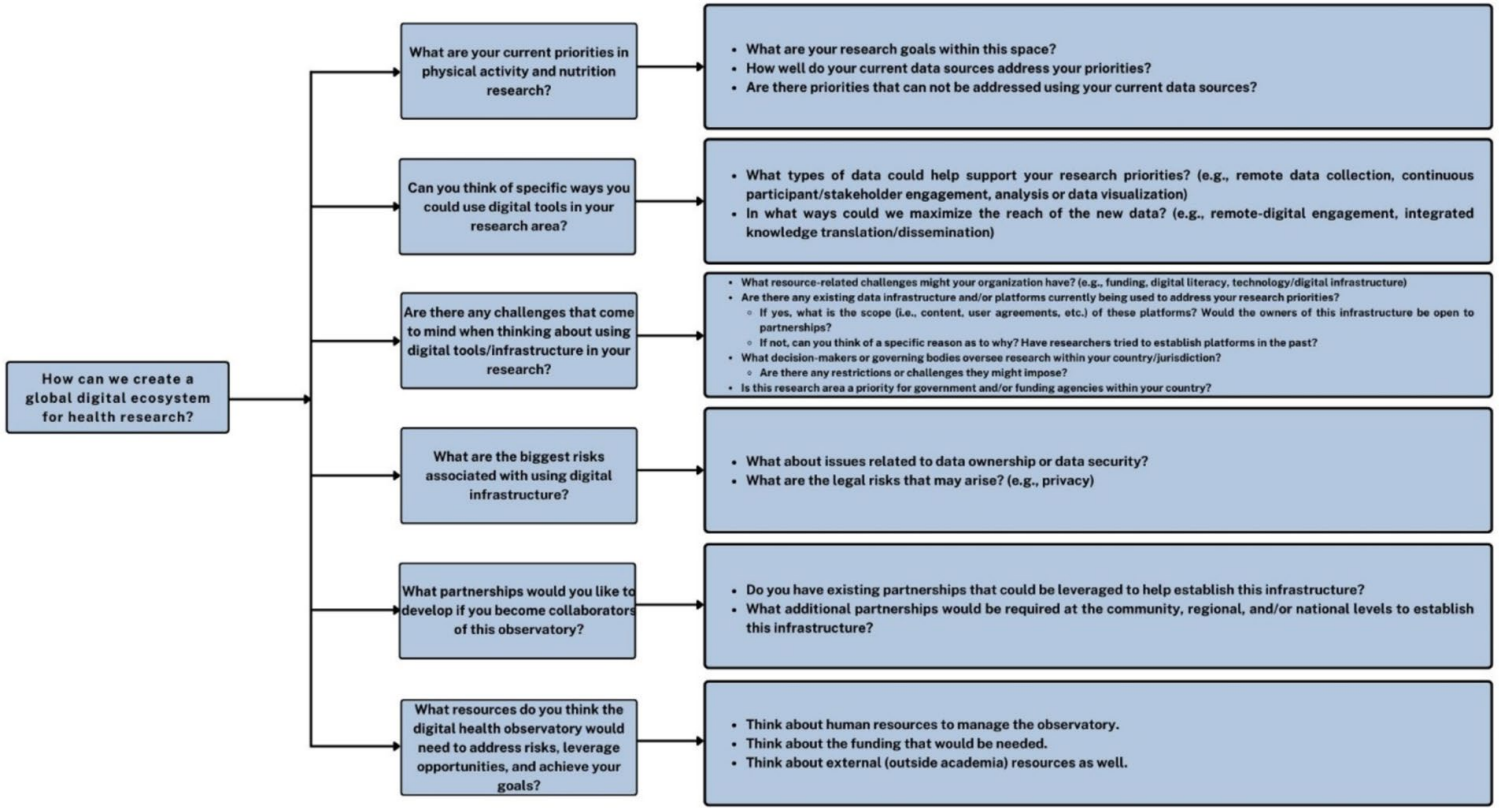
Systems mapping guide.

Thereafter, after addressing stakeholder queries about DiScO, Moderator 3 explained the concept and rationale for real-time systems mapping, a methodology which aimed to create a global digital ecosystem for health research by tracing connections to pinpoint crucial intervention points. Stakeholders were given a detailed explanation of the systems mapping activity, which was developed by the moderators who have expertise in stakeholder engagement, digital evaluation, and systems mapping. The systems map created in real-time by the moderators incorporated content from both independent group discussions and the overall group discussion. In essence, stakeholder discussions were visually represented in real-time on a smartboard using a digital collaboration platform (Miro Inc. 2011, Amsterdam) to create a rapid systems map based on the themes relayed by the stakeholder groups (Figure 3).

**Figure 4:**
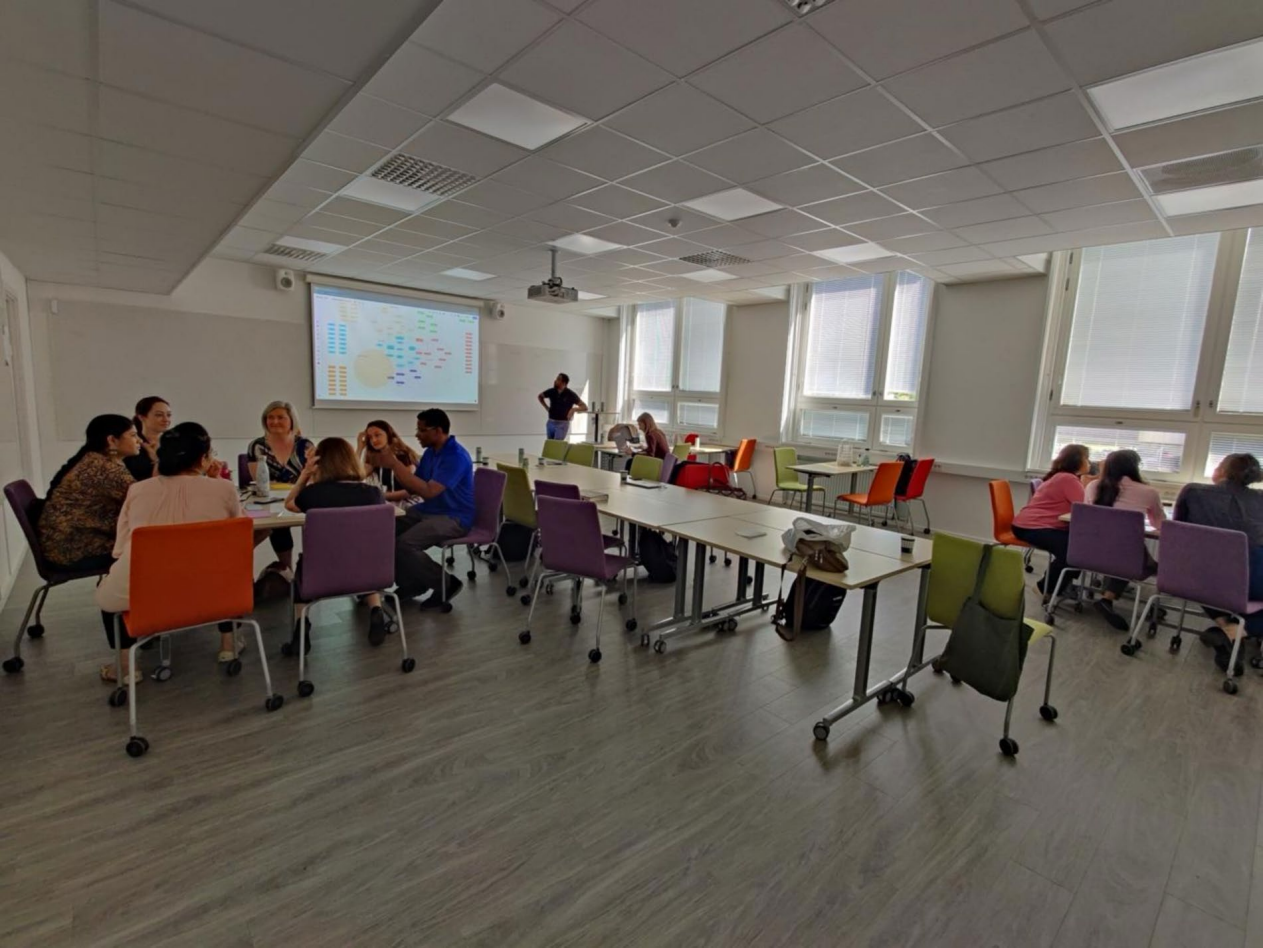
Group discussions relayed in real-time to develop rapid systems map.

Stakeholders were randomly divided into two groups and were provided with a systems mapping guide (Figure 4) developed by the moderators by adapting an evidence-based needs assessment framework to embed digital health platforms across jurisdictions (37). The framework was developed for identifying jurisdictional priorities that can be addressed by developing and implementing digital platforms. The tool was adapted and applied to engage stakeholders from low-, middle-, and high-income countries with a focus on developing DiScO health research. Stakeholder group discussions focused on the six categories (i.e., themes) captured in the systems mapping guide: priorities, opportunities, challenges, risks, partnerships, and resources. Stakeholders were asked to consider six key questions, and corresponding prompts, to guide their discussions. Stakeholders spent 50 min in two groups (Figure 4) discussing the potential implementation and operationalization of DiScO across the six pre-identified systems map themes, all of which were given equal consideration during discussions. Each group self-assigned a team leader to take notes of the key points emerging from themes, which were displayed on a segregated white board (groups 1 and 2) using sticky notes.

These key points were relayed by Moderators 1 and 2 to Moderator 3, which enabled them to create a rapid systems map in real-time. All stakeholders were brought together for a final discussion, where the preliminary rapid systems map was revealed on a large monitor and was subject to real-time modifications by Moderator 3 through an overall discussion (including all stakeholders) guided by Moderators 1 and 2. The overall group discussion was conducted to elicit corroborations, corrections, and further suggestions, where all stakeholders had a chance to share their perspectives and challenge notions. Mitigation strategies were also brainstormed as a group to overcome the identified risks and challenges. The rapid systems mapping ended with a general consensus that all stakeholder voices were captured.

### 2.4 Data analyses

The three audio recorded files (2 independent group discussions and 1 overall discussion) from the systems mapping session were transcribed and thematically analyzed by two independent analysts using NVIVO 14 software. A coding manual was created, using the most common words and phrases to create a number of “nodes.” The analysts then reviewed the nodes for significant overlap to create themes and subthemes. The analysts compared their findings to reach a consensus through a structured analysis process, and selected the most relevant and impactful quotations from each theme/subtheme (see Appendix A) (37). Quotations were captured and labeled according to their respective discussion groups and represented multiple voices – overall group discussion (OGD), independent group 1 discussion (G1), and independent group 2 discussion (G2). These themes were further validated by a literature review, whereby existing and new interconnections between theme categories were corroborated with evidence. The purpose of this analysis was to thoroughly examine key themes across all session discussions, ensure internal validity of our study approach, and refine the preliminary rapid systems map to develop a revised comprehensive rapid systems map grounded in empirical evidence. This internal validation process follows established systems mapping methods, which includes document analysis (i.e., qualitative analysis of transcribed data from two separate groups of stakeholders) to internally cross-validate and triangulate data to increase validity of the results (45).

## 3 Results

**Figure 5:**
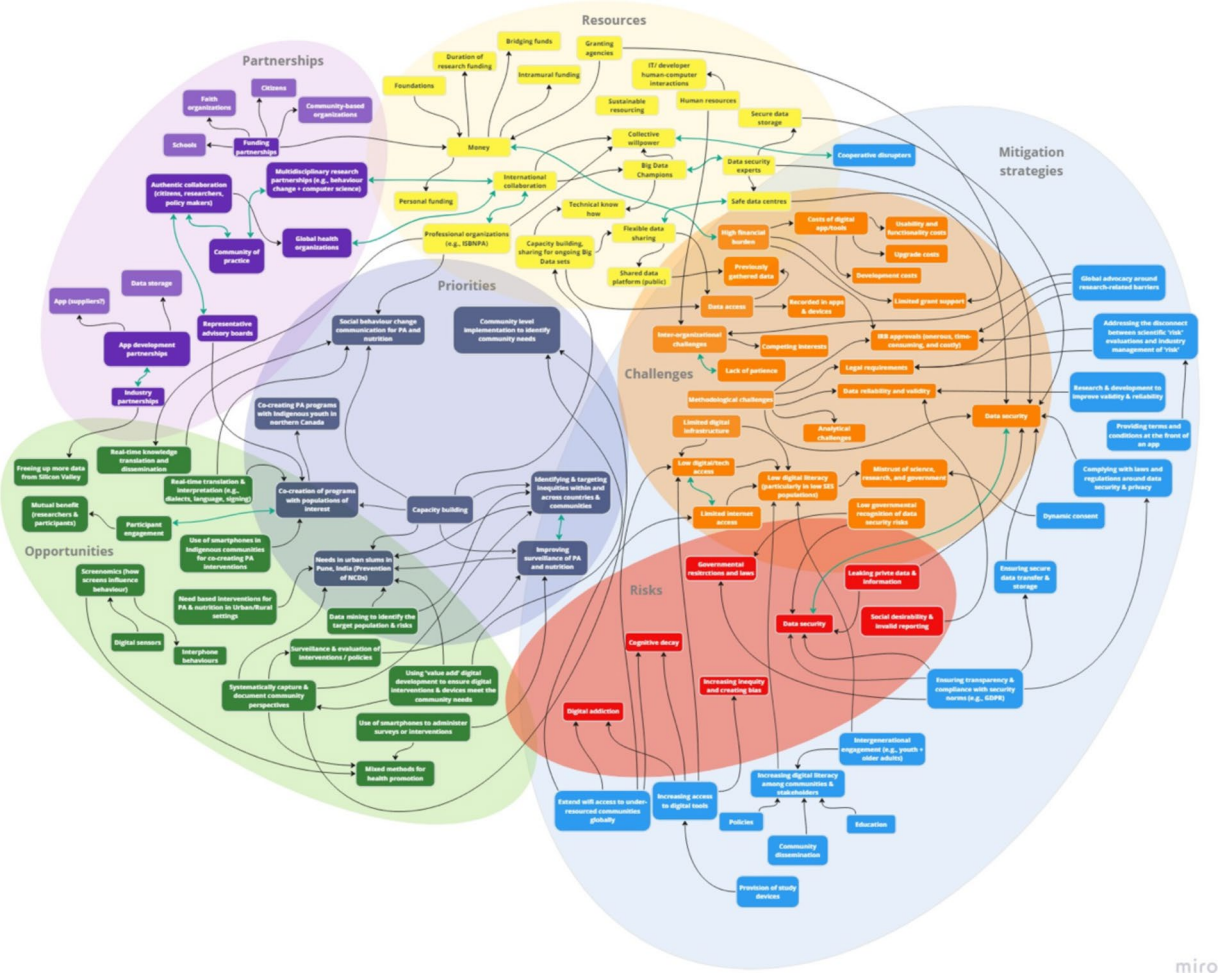
Rapid systems map created in real-time (pre-analysis).

**Figure 6:**
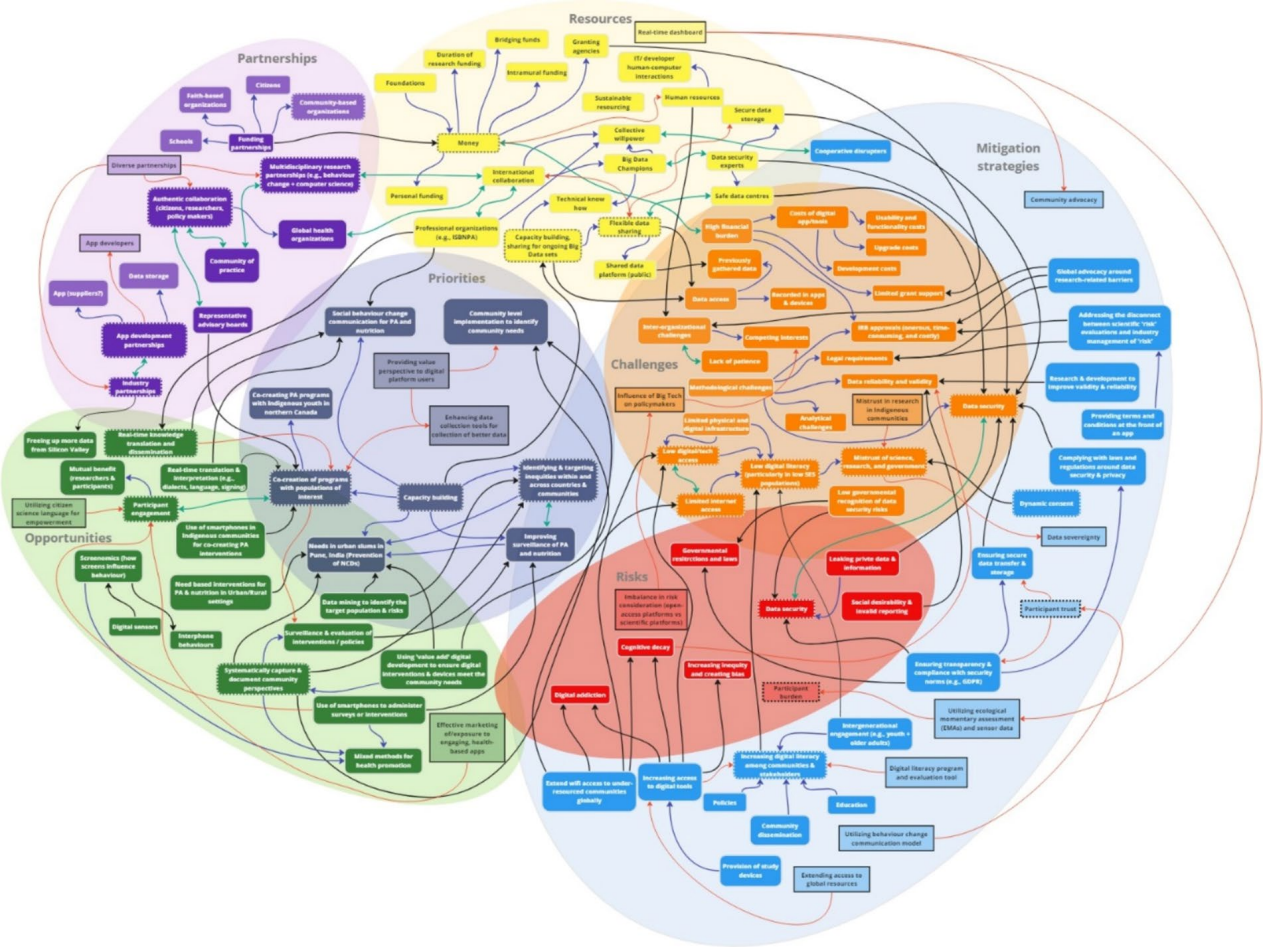
Adapted rapid systems map (post-analysis).

The study resulted in the development of a rapid systems map (Figure 5), which was further adapted (Figure 6) based on evidence generated from the international stakeholder discussions. The adapted rapid systems map (i.e., after data analyses) depicts a total of six themes from the rapid systems mapping guide (priorities, opportunities, risks, challenges, partnerships, and resources) and 23 key nodes of intervention. Additionally, a new theme emerged organically through stakeholder group discussions – mitigation strategies – with 3 corresponding nodes of intervention.

In the pre-analysis rapid systems map (Figure 5), within and between theme unidirectional (black arrows) and bi-directional (green arrows) links were found indicating relationships between key factors (enumerated in rounded squares within themes). These links were created and visualized in real-time by utilizing stakeholder discussion points.

The pre-analysis rapid systems map that was created in real-time was validated with a post-analysis rapid systems map after analyzing the recorded stakeholder discussions (Figure 6). The thematic analysis enabled our team to enrich the rapid systems map by capturing nuanced discussion points that may have been overlooked in real-time by introducing some additional key factors (enumerated in squares within themes).

Overall, new factors were introduced in all seven themes of the post-analysis systems map that not only depicted intra (blue arrows), but also inter-thematic links (black arrows). Most importantly, none of the key factors in the pre-analysis rapid systems map were deleted to corroborate real-time findings. Thus, the findings that substantiate the key factor introduction, visualization of intra and inter-thematic links, and the depiction of key nodes of intervention (enumerated with dotted borders) only within the post-analysis rapid systems map are presented below by categorization of themes into sub-themes based on evidence from independent and overall group discussions.

### 3.1 Theme 1: Priorities

Analysis of stakeholder discussions about ‘priorities’ revealed key subthemes, which substantiated the key factors that were identified in real-time during the systems mapping exercise: capacity building, co-creation, equity considerations, targeted interventions for NCDs, and a focus on community-level perspectives. Two key factors were added to the systems map after data analysis to reflect the comprehensive discussions: value perspective for citizens and enhancing data collection tools. Stakeholders discussed the significance of providing tangible value for users to use digital health platforms. An example given by session facilitators was the willingness of users to provide personal data to Google in return for full access to their Map application features. During discussions, the concept of value perspective was connected to the need to enhance digital data collection tools through the co-creation of surveys and programs with specific communities. In addition, “enhancing data collection tools for collection of better data” was added and connected to the factor “value perspective.” “Data collection tools” was also connected to the existing subtheme “Co-creation of programs with populations of interest.” Co-creation and community input was seen as a valuable resource to facilitate “Surveillance & evaluation of interventions/policies” – an inter-thematic connection between Priorities and Opportunities.

**Figure 7:**
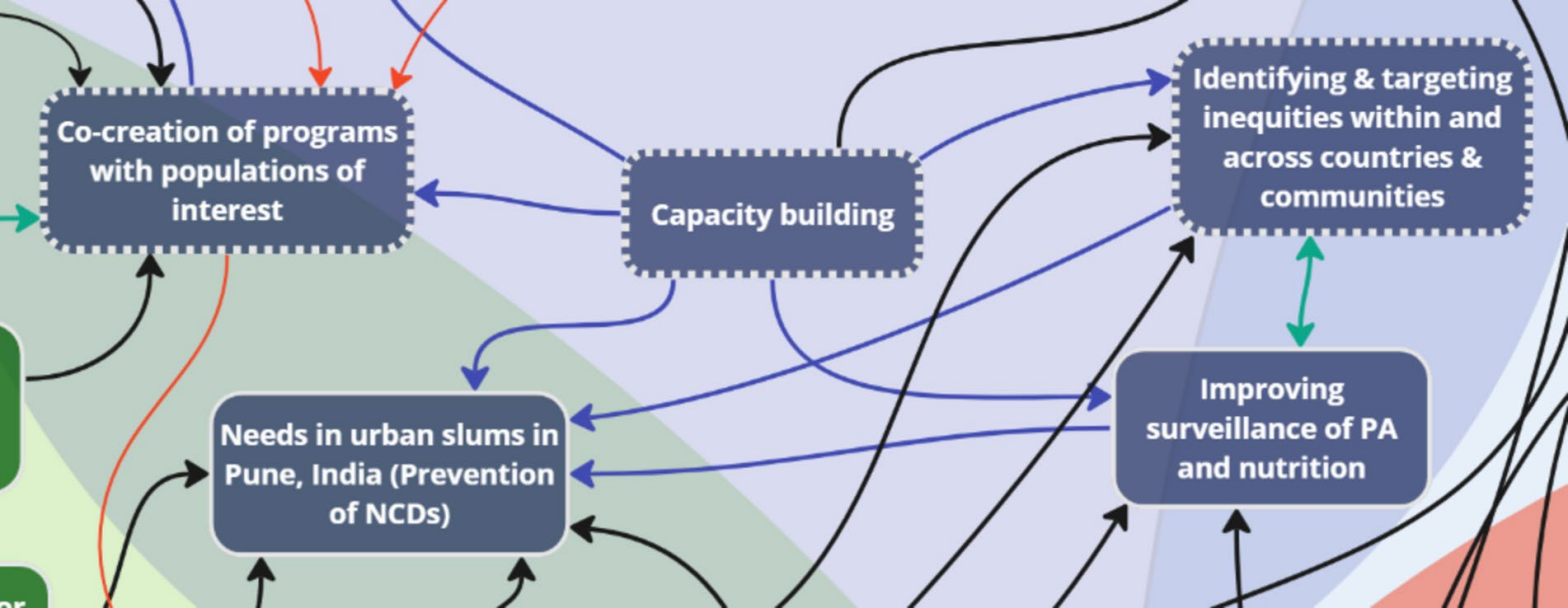
Key nodes of intervention within ‘Priorities’.

More importantly, three key nodes of intervention were identified with the priorities (Figure 7), which formed an interdependent relationship with the context of addressing common challenges: *capacity building, co-creation, and equity.* Capacity building across sectors and countries was emphasized due to jurisdictions having varying capacities, which requires sharing resources, expertise, and technology. For instance, stakeholders noted that:

*“Because we need to have a better collaboration between researchers and policymakers. But communities as well, and across different levels, because we know that the needs and the priorities are the same at different levels of community level. There’s more collaboration and more, like, sharing resources and expertise.”* (OGD)

Capacity building was connected with the value of co-creating programs with specific communities to address local priorities. One stakeholder noted:

*“Co-creating physical activity programs with Indigenous youth in Northern Canada. We can also extend this point to say co-creating any such program with the community that you’re looking to serve in your respective jurisdiction, of course.”* (OGD)

Also linked to capacity building was the potential of using smartphones as tools for equity due to their widespread accessibility, with one stakeholder stating:

*“You know, I’ve been asked this question, “why are you so focused on smartphones?” Because I don’t care about smartphones. I was a late adopter of a smartphone. But what interests me is its market penetration, and it could be a tool of equity because everyone’s got it.”* (OGD)

The challenge of ensuring equitable access to technology was also touched upon by one stakeholder, acknowledging disparities in internet access in smaller communities:

*“The interesting thing is lack of equitable access to Internet is an issue. When we go to smaller communities, we see kids grouping around libraries or McDonald’s, which is an interesting intersection with nutrition altogether because they’re getting free Wi-Fi, right?”* (OGD)

The findings indicated that by building capacity, engaging in co-creation with communities, and prioritizing equity, stakeholders can work toward more effective and sustainable interventions that promote health and wellbeing globally. The full list of subthemes and supporting quotes can be found in Appendix A.

### 3.2 Theme 2: Opportunities

Key subthemes from stakeholder discussions on the topic of ‘opportunities’ included: engagement, knowledge translation and dissemination, community input, screen omics, and data mining. Post-session analysis introduced two additional key factors to the systems map: utilizing citizen science language for empowerment, and effective marketing of health-based apps. Stakeholders emphasized the importance of language to balance power dynamics and increase involvement of participants. During discussions, stakeholders came to a consensus on the need for engaging health-based apps through marketing strategies used in other (i.e., non-health-related) industries to effectively attract users. Both factors added were linked to engagement, as they may influence participants’ level of interest and engagement in a study.

**Figure 8:**
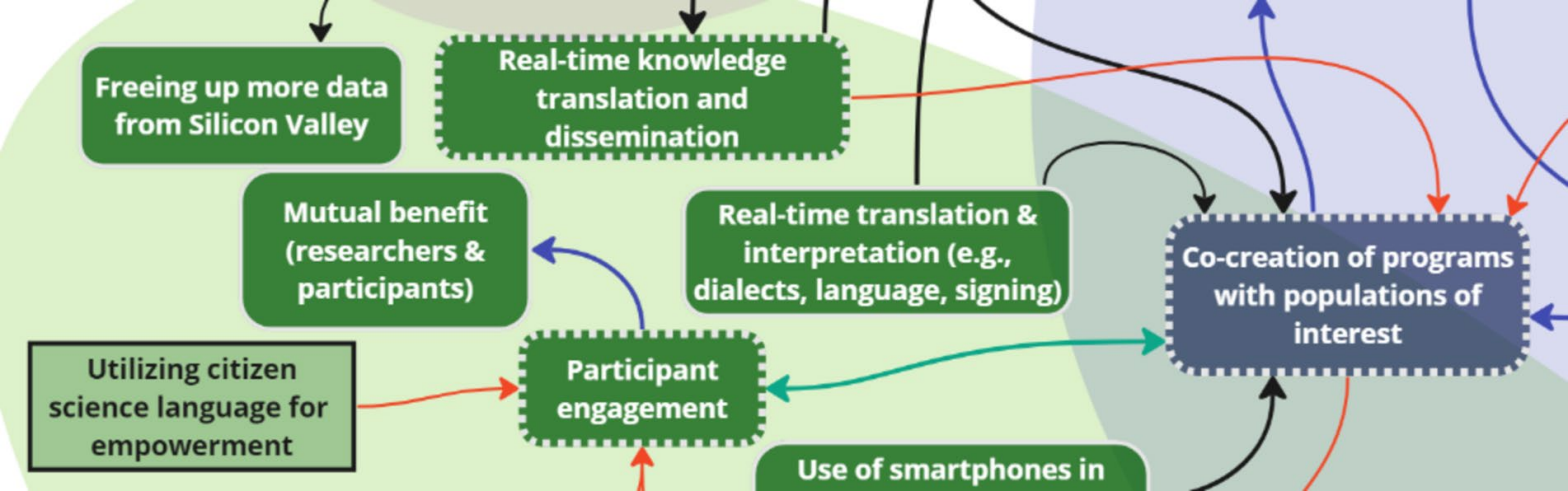
Two key nodes of intervention (engagement and knowledge translation and dissemination) within the theme ‘Opportunities’.

**Figure 9:**
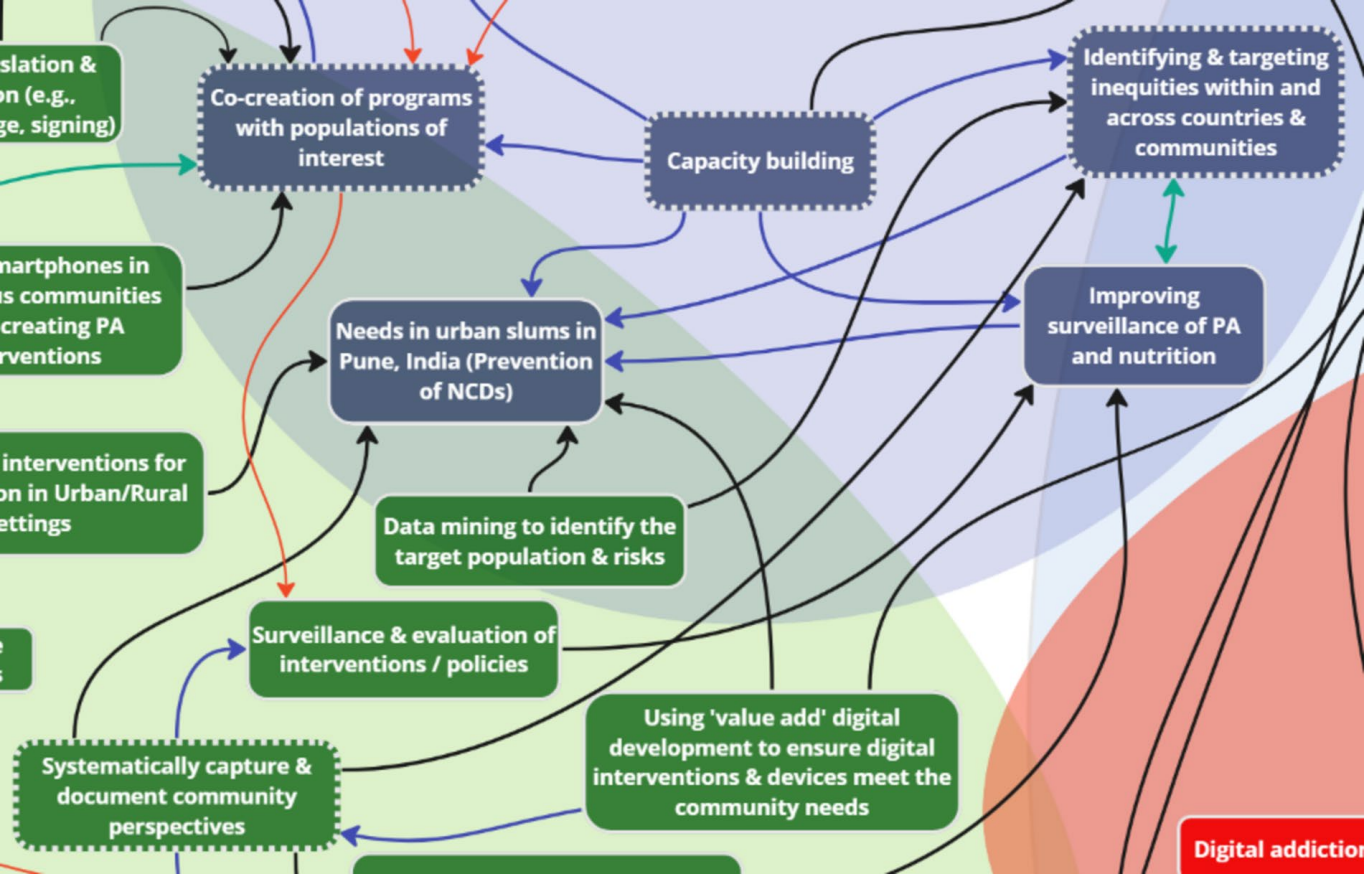
One key node of intervention (community input) within ‘Opportunities’.

For opportunities, three key nodes of intervention were identified to maximize the reach of new data: *engagement, knowledge translation and dissemination, and community input* (Figures 8, 9). One stakeholder discussed the potential of using smartphones in communities for engagement without reliable network access, recognizing that despite connectivity challenges, almost everyone has a smartphone:

*“Because the majority of the populations that I work with are in what are known as reserves, so they live on reserve, which is communities that are further North in Canada … but everybody has a smartphone, so smartphones could be a way to create this.”* (G1)

Some stakeholders also emphasized the potential for mutual benefit in engaging participants:

*“So, it’s engagement of target participant and mutual benefit of both researcher and the participant.”* (G2)

Engagement was bi-directionally linked to a ‘priorities’ key node of intervention: co-creation. Another inter-thematic connection was added following the analysis. The connection was between knowledge translation and dissemination (opportunities), and co-creation (priorities) to depict the importance of user involvement and collaboration in designing and implementing initiatives for the specific needs of target communities. Multiple stakeholders suggested the use of visual aids like graphs and charts for effective dissemination of findings and accessibility to big data for decision-making:

*“Real-time translation and dissemination of knowledge, especially when you’re using something with a dashboard system which is able to collate statistics and present them graphically or visually. This is something that is a real advantage of being able to do KT in real-time.”* (OGD)

Community input, which was inter-thematically linked to equity – a ‘priorities’ key node of intervention – was seen as a valuable resource for surveillance and evaluation of interventions and policies, with some stakeholders stating:

*“If you want to do good surveillance, and if the digital literacy is low, it’s a no brainer that we won’t be able to do it good surveillance, or ethical surveillance.”* (OGD)

Additionally, the idea of identifying key individuals within communities, especially youth equipped with smartphones to serve as representatives, was mentioned as another potential avenue for leveraging community input in intervention and policy evaluation:

*“Or the other way could be identifying like key, youth – youth within each community and have each one with a smartphone for example, and they could then become the champions within their communities.”* (G1)

Prioritizing participant engagement, effective knowledge translation and dissemination, and community input can enhance the uptake of research initiatives as indicated by the stakeholder discussions. Appendix A contains a comprehensive list of subthemes and supporting quotes.

### 3.3 Theme 3: Challenges

Stakeholder discussions revealed several key ‘challenge’ subthemes: competing interests, costs, digital/internet access, digital literacy, mistrust, validity and reliability of data, big tech, lack of resources, and privacy and data security. An additional two key factors were introduced to the systems map after analysis: mistrust in research in Indigenous communities and influence of big tech on policymakers. Stakeholders discussed that a history of colonization (in the North American context) was connected to “mistrust of science, research, and government” among Indigenous communities due to mistreatment by entities in power. In addition, one group discussed the challenge of big tech due to their influence on the political environment, which was intra-thematically linked to ‘Competing interests’ and inter-thematically linked to ‘Data sovereignty,’ a key factor within mitigation strategies.

**Figure 10:**
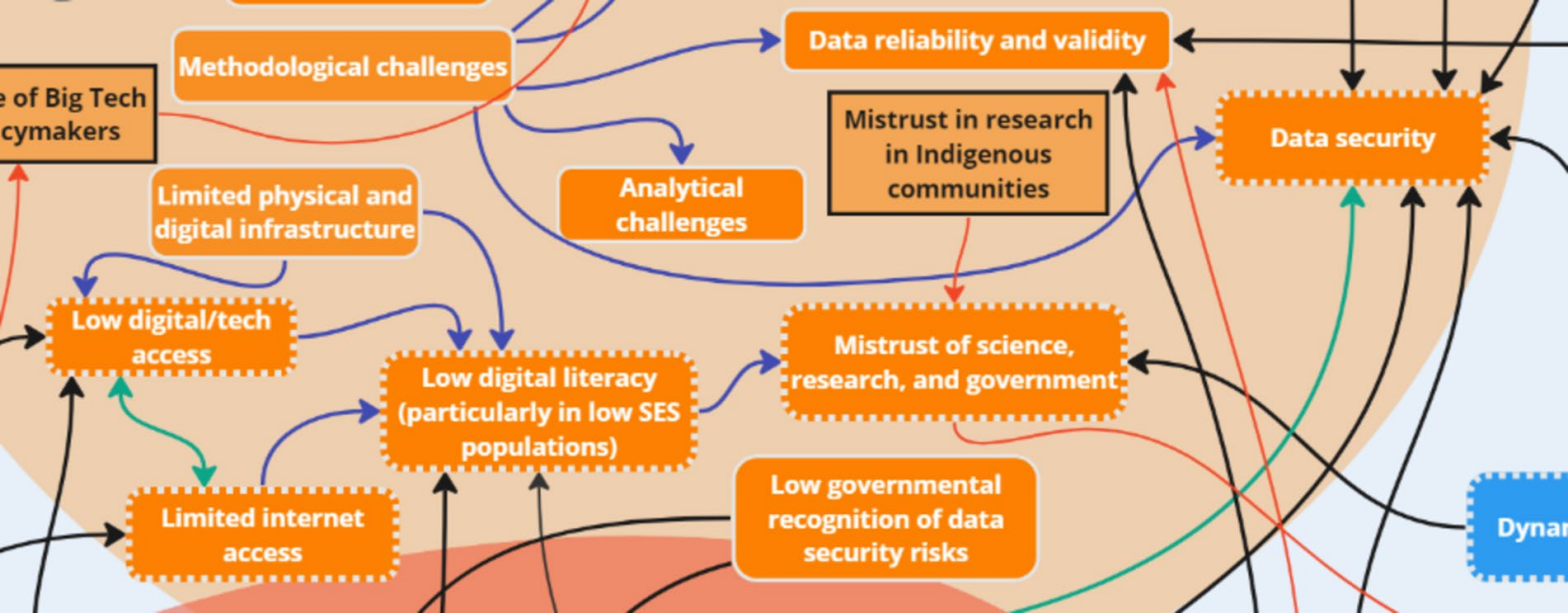
Key nodes of intervention within the theme ‘Challenges’.

Following analysis of stakeholder discussions, the following key nodes of intervention were identified within the challenges: *digital/internet access, digital literacy, mistrust, and privacy and data security* (Figure 10). Discussions between all groups (OGD, G1, G2) highlighted challenges related to digital access and internet access – two key factors that were bi-directionally linked to one another. One stakeholder stated:

*“Yeah, access could be low, internet access could be low. It's huge. Without internet, nothing functions.”* (OGD)

Issues such as low internet connectivity, limited Wi-Fi access, and lack of digital technology access were emphasized in under-resourced communities:

*“…for example, in Canada, really, I think it's criminal […] like I go to a community that's literally 300 kilometers from a major center, and there's zero connection zero. Like there's no phone network, there's like you're 300 kilometers away from a major center. Just to me, it is just because the people that live there, there is – they're not a priority to them…”* (G1)

The impact of these challenges on data collection in rural areas, as well as concerns about the neglect of communities without proper connectivity were expressed, with stakeholders highlighting inequities that may arise due to differential access:

*“So increasing inequity and bias [are challenges] because if people have less access, they are really already like facing inequities and […] because now that everything is getting digital, they're probably face even more restricted.”* (G2)

Digital access and internet access were both linked to low digital literacy, highlighting the significant challenge of varying digital literacy in health research, especially among low socioeconomic status groups:

*“I see digital literacy, particularly in low [socioeconomic status] populations. This is certainly a huge issue and a huge barrier because even if someone has a smartphone, it doesn't mean that they're able to engage with an app that we've developed or use it in a way that maybe is as useful as it could be to them, depending on their digital literacy level.”* (OGD)

Connected to the factor ‘low digital literacy’ was the issue of ‘mistrust in health research,’ with stakeholders stating:

*“So the point I would add is variation of mistrust across jurisdictions. Yes, every jurisdiction has its own issues -- with mistrust that could be a big challenge.”* (OGD)

Concerns also included the harmful legacy of colonization in Indigenous communities, highlighting the importance of data sovereignty in digital health apps:

*“So the history of Indigenous communities is – there's a history of Western researchers stealing data or taking data not giving anything back, all sorts of things have happened in the past. So the idea of – the concept of sovereignty is very, very important […] Because we're saying, ‘Who owns the data?’ and Indigenous communities would like to own their data, right? They value – and data sovereignty is big these days. Data is almost everything, right?”* (OGD)

Stakeholders also mentioned concerns related to privacy and data security. While efforts are made to maintain anonymity, the emphasis on global positioning systems and geocoding introduces potential risks, especially regarding the misuse of location data:

*“Geocoding has to be also decentralized […], otherwise they can use the geocoding with the timeline to analyze your work daily working pattern…”* (G1)

These findings indicate that addressing the challenges of low digital/internet access, which is linked to low digital literacy, and further perpetuates mistrust in science is necessary when implementing digital infrastructure into research initiatives. Additionally, data and privacy concerns are crucial for ethical participation. The full list of subthemes and supporting quotes can be found in Appendix A.

### 3.4 Theme 4: Risks

Stakeholders identified key subthemes related to ‘risks,’ including: cost–benefit, risk to science, data inaccuracy, excess screentime/digital addiction, and increasing inequity. Participant burden and imbalance in risk consideration (open-access platforms vs. scientific platforms) were two key factors that were added to the initial map following analysis. Stakeholders discussed the potential risk of participant burden with continuous mobile surveys. They also discussed risk considerations related to the use of open-access platforms vs. scientific platforms, specifically the disconnect between research-based versus open-access platforms which were perceived to require relatively less rigorous protocols for data collection. This was intra-thematically connected to the challenges subtheme “Influence of Big Tech on policymakers,” which does not aid the scientific community utilizing digital resources.

**Figure 11:**
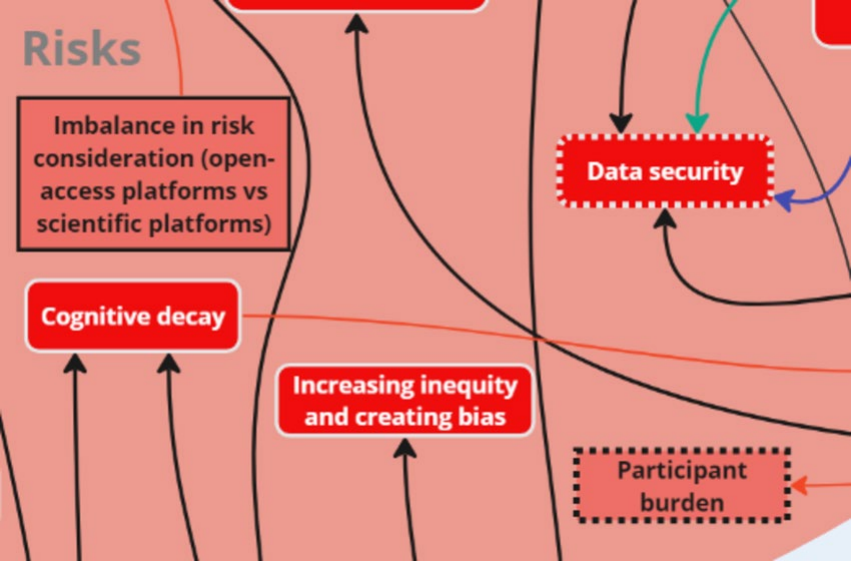
Key nodes of intervention within the theme ‘Risks’.

Two key nodes of intervention were identified with the risks: *cost–benefit* and *participant burden* (Figure 11). The risks mentioned above, i.e., privacy concerns and government regulations, contributed to stakeholders’ assessment of the overall costs and benefits associated with utilizing digital apps for mental health improvement. One stakeholder noted:

*“…leaking private data, government restrictions and laws, increasing inequity and creating bias, of course, addiction – that’s an interesting one because this is something that comes up a lot when we are even talking about physical activity […] or mental health research where if we are using a digital app to help improve youth mental health, but we know that smartphones are also especially used for social media [which] is contributing to poor mental health, where’s the cost–benefit there, right?”* (OGD).

Stakeholders also discussed how participants may experience burden from continuous mobile surveys, leading to feelings of overwhelm or fatigue. Stakeholders offered innovative approaches, such as ecological momentary assessments (EMAs) and sensor data, to address this issue:

*“…we have been using something called ecological momentary assessments to make the process of completing what feels like a survey, but should not feel like a survey, a lot more expedient […]. So that’s something that we also are actively working on as part of this to decrease the fatigue and increase the actual completion of some of this data collection.”* (OGD).

As key nodes of intervention within ‘risks’ suggest, improving cost–benefit and participant burden can be concurrently addressed for more effective health interventions. A comprehensive list of subthemes and supporting quotes can be found in Appendix A.

### 3.5 Theme 5: Partnerships

Stakeholders discussed the theme of ‘partnerships’ with a focus on industry partnerships, organizations, developers and computer scientists, and citizens and communities. Two key factors were added to the systems map to comprehensively capture stakeholder discussions: diverse partnerships and app developers. Diverse partnerships were connected to multiple existing factors, including “Authentic collaboration (citizens, researchers, policy makers),” “Multidisciplinary research partnerships (e.g., behavior change + computer science),” and “Industry partnerships” to emphasize the need for diversity of partnerships across disciplines and sectors. App developers was also added and connected to “App development partnerships,” as stakeholders emphasized the specific need for these partnerships to build digital infrastructure.

**Figure 12:**
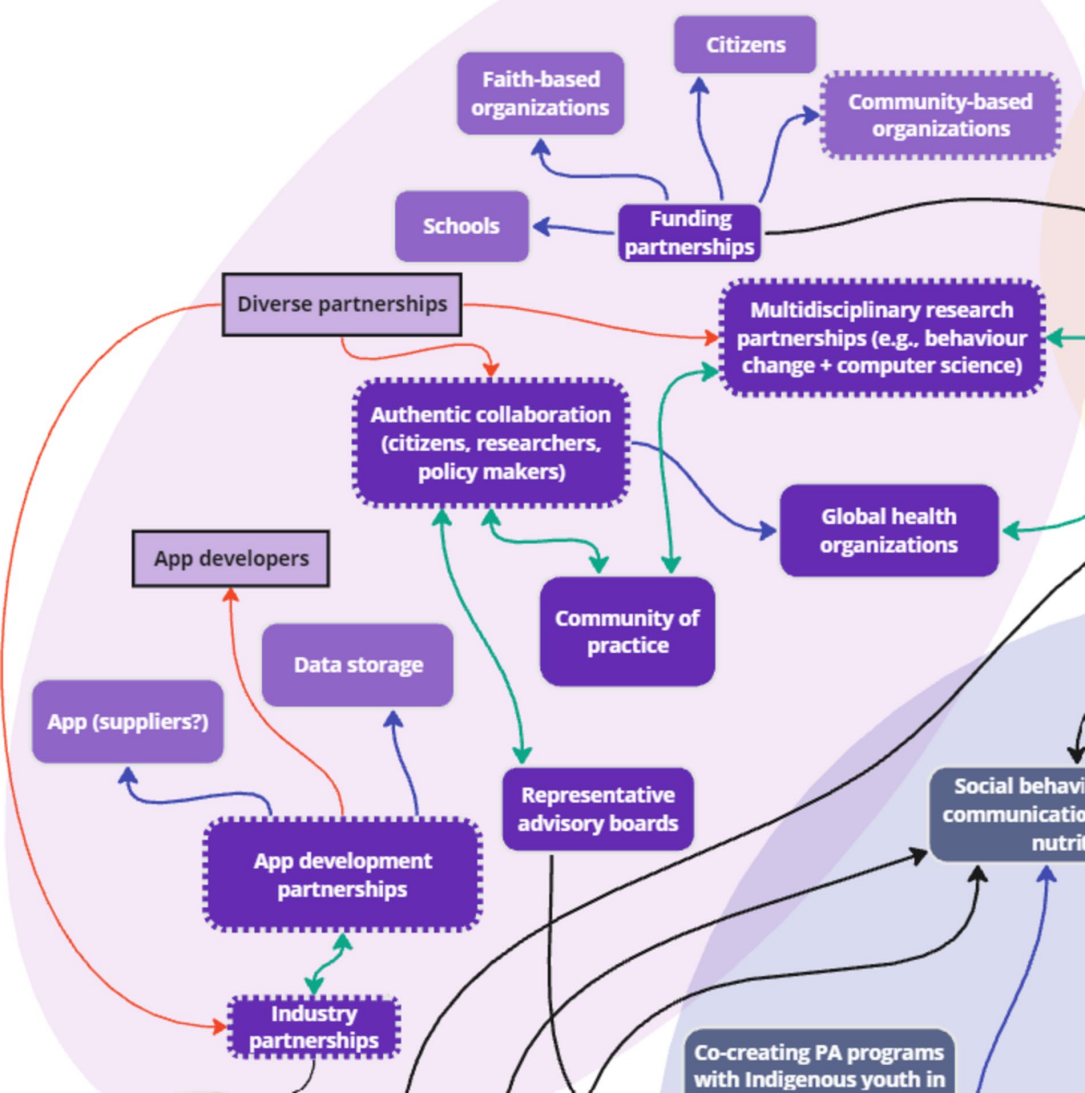
Key nodes of intervention within the theme ‘Partnerships’.

In addition, four key nodes of intervention were identified in partnership development: *industry partnerships, developers and computer scientists, citizens and communities, and organizations* (Figure 12). Stakeholders discussed the need for industry partners to help move research projects forward for real change, noting:

*“The partnership with the industry emerged in our table as more important. Maybe because without including them in the boat, then we can just do research and then what is the change if they are not included?”* (OGD).

Industry partnerships were linked to developers and computer scientists, who work in different industries than these stakeholders with skills specifically in data storage and ongoing support:

*“Yeah, I said partnerships with somebody who has the understanding of the app development, who can do what we are asking them because I can give you the idea, but I have no understanding of how that app is developed, what goes on in the background, and then once that app is developed to sustain supported, that would be something –.”* (G1).

They also highlighted the importance of collaboration with researchers from scientists across disciplines such as human-computer interaction sciences, computer sciences, and behavioral sciences:

*“So, among researchers, so it could be researchers that are good in computer science or researchers who are in behaviour sciences and behavioural research.”* (G1).

Stakeholders stressed the importance of closely collaborating with communities in managing and utilizing data collected to ensure equal collaboration through authentic partnerships, which could be formed using participatory approaches such as community advisory boards:

*“… I always talk about this, like an authentic partnership between and amongst our researchers [and] policy markers at every level and the citizens themselves, so as equal partners, right, not just the talk not like the hierarchy, yes, as equals, so that everybody feels like they can be heard.”* (G1).

*“They also called advisory councils where you have representation of different stakeholders. And in the case of a community of practice. It could be practitioners. It could be actual community members who come together to give that type of feedback about how for address something because they are representatives of their respective groups or respective disciplines, and they are able to share that in a more community advisory boards.”* (G2).

The findings indicated that building partnerships with diverse industries and development teams, as well as authentic collaboration with citizens, communities, and organizations is necessary for developing effective and usable digital infrastructure for health research initiatives on a global scale. The full list of subthemes and supporting quotes can be found in Appendix A.

### 3.6 Theme 6: Resources

During the discussion regarding ‘resources’ required to implement digital health interventions, stakeholders identified the following key subthemes: data sharing, funding, big data, data storage, and real-time dashboard for advocacy. One key factor – “Real-time dashboard” – was added as a resource and inter-thematically connected to the mitigation strategies “Community advocacy” and “Utilizing EMAs and sensor data.”

**Figure 13:**
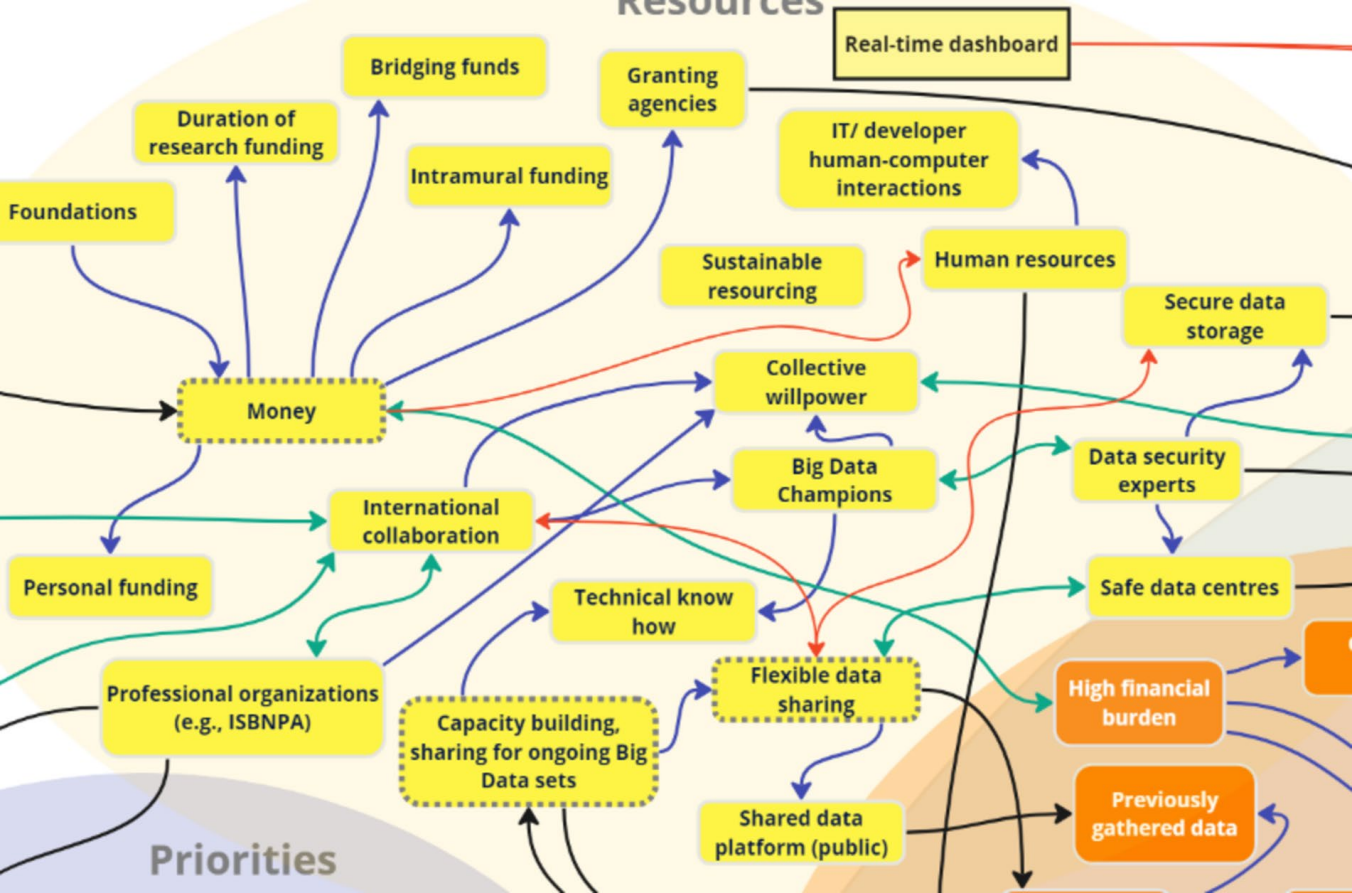
Key nodes of intervention within the theme ‘Resources’.

Three interlinked key nodes of intervention were identified following analysis of stakeholder discussions about resources that the digital health observatory would need to address risks, leverage opportunities, and achieve goals: *funding, data sharing, and big data* (Figure 13). The most commonly discussed resource across discussion groups (OGD, G1, G2) was financial resources for research project implementation, with one stakeholder stating:

*“So some of the resources – money, money, money, no doubt. No doubt about that. Yeah. And that’s something that I think once we start to making interconnections, that bubble would be bigger and bolder, because you cannot really do anything without it.”* (OGD).

A related recurring theme was the essential role of financial and human resources, which are linked on the map, in overcoming obstacles and ensuring the longevity of research initiatives, with stakeholders stating:

*“I think both your points are very good in addressing what OGD said. Many times, we have a long-term program and for us, like, they fund on year-to-year basis, and we are like, at the end of the year, where are we supposed to go? We cannot stop the research to wait until we get more money. We do not have enough fundings to keep the research going, but that’s a huge problem.”* (G1).

In addition to funding, stakeholders expressed interest in flexible data sharing and data platforms as a resource, highlighting the importance of public, secure data storage, and international collaboration. Additionally, the idea of a digital dashboard for sharing local-level data was also mentioned:

*“…I mean the idea that we would have a dashboard where everybody [could see data]. Yeah, local level data and then you could look – all the partners could [see] all the [data], you know, people on the network could look up walkability projects from communities around the world.”* (G2).

Stakeholders recognized that data sharing can only happen when data is available, which is why it was linked to big data for capacity building. Specifically, the concept of big data and its implications were discussed, with the mention that big data does not necessarily require a large sample:

*“So, you alone are generating big data everyday. Big data does not mean it comes from 100,000 people. Big data can come from one person themselves, because the velocity at which you are generating data, because you own a smartphone or a device, is incredible.”* (G1).

The findings indicate that no project implementation is possible without proper funding, which can then facilitate other essential resources such as data sharing of big data sets for capacity building. A comprehensive list of subthemes within resources can be found in Appendix A.

### 3.7 Theme 7: Mitigation Strategies

While ‘mitigation strategies’ was not one of the initial themes presented by facilitators to stakeholders during the session, two of the three discussion groups organically mentioned mitigation strategies in relation to the challenges and risks discussed. The key factors revealed through real-time discussions were further corroborated following thematic analysis of discussions and identification of the following key subthemes: dynamic consent, data security, and digital literacy. Seven key factors were added to the systems map following analysis of stakeholder discussion: participant trust, utilizing EMAs and sensor data, digital literacy program and evaluation tool, utilizing behavior change communication model, extending access to global resource, data sovereignty, and community advocacy.

“Utilizing behaviour change communication model” and “Participant trust” were connected to address potential participant hesitancy to engage in digital research projects, and to spread knowledge effectively, thus gaining the trust of research participants. “Participant trust” was also found to be related to “Ensuring secure data transfer & storage” and “Ensuring transparency & compliance with security norms (e.g., General Data Protection Regulation).

One stakeholder discussed the creation of a “Digital literacy program and evaluation tool,” which was connected to the existing key factor “Increasing digital literacy among communities & stakeholders.” “Increasing access to digital tools” was perceived to increase digital literacy, thus this connection was made on the map. “Extending access to global resources” was seen as another mitigation strategy to stakeholders, which was associated with “Increasing access to digital tools.” “Utilizing EMAs and sensor data” was a mitigation strategy added and inter-thematically connected to the “Participant burden” risk.

**Figure 14:**
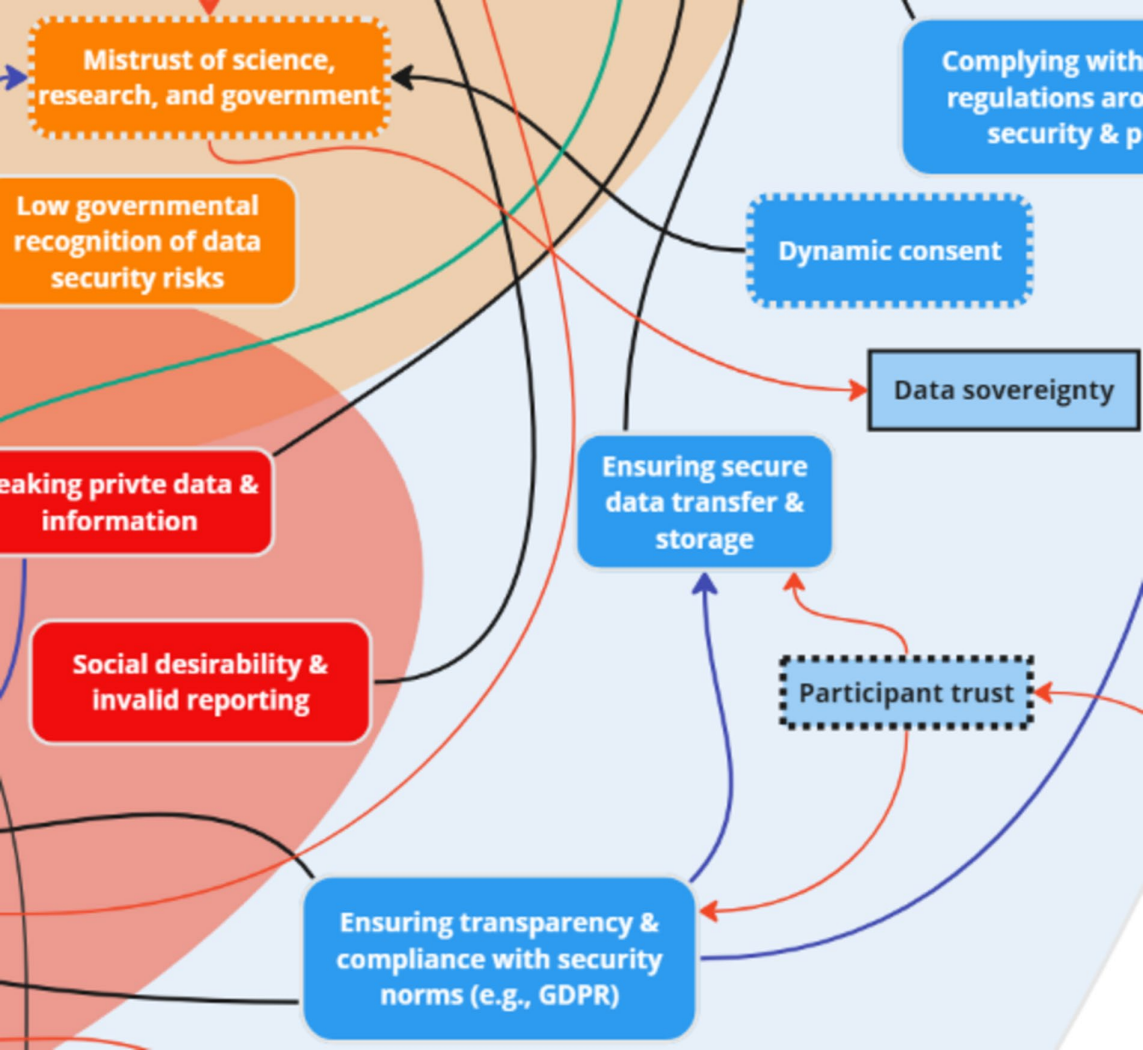
Two key nodes of intervention (dynamic consent and trust) within the theme ‘Mitigation Strategies’.

**Figure 15:**
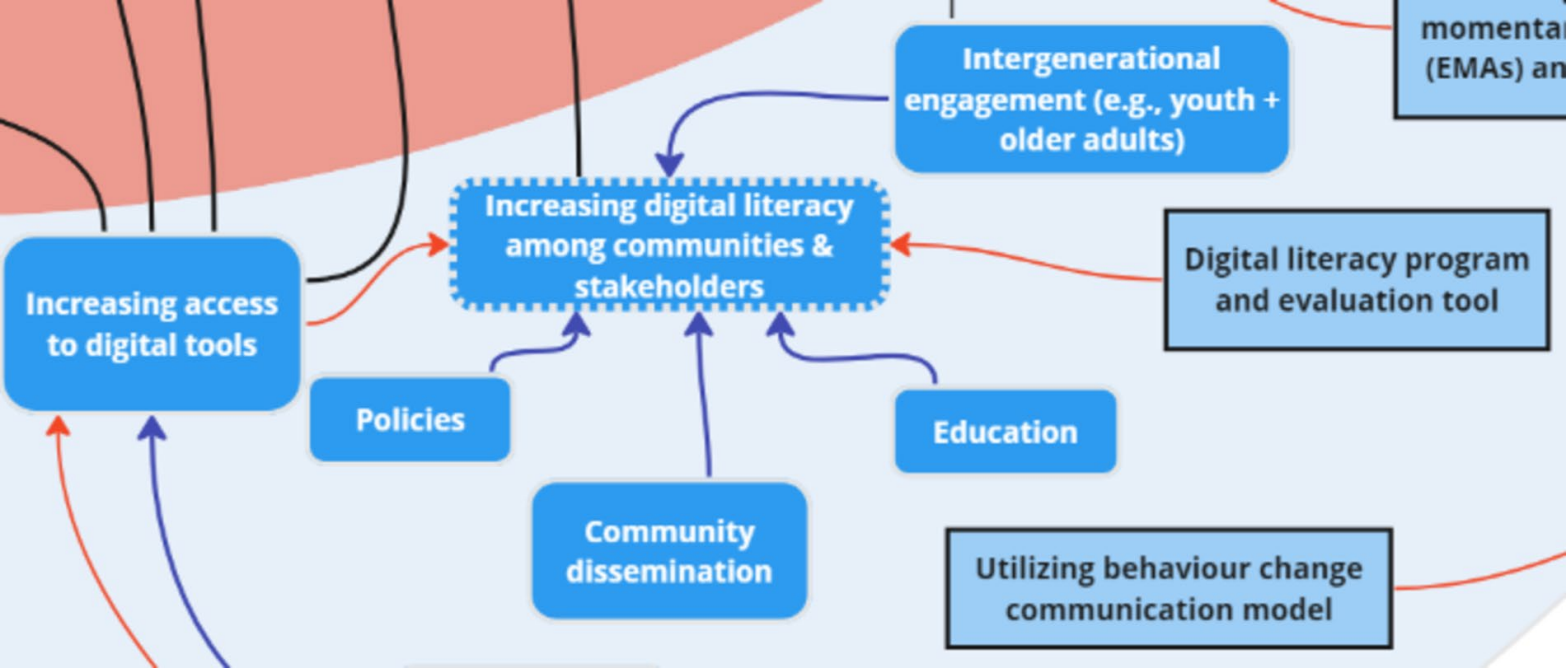
One key node of intervention (digital literacy) within the theme ‘Mitigation Strategies’.

Four key nodes of intervention were identified following analysis of stakeholder mitigation discussions: *dynamic consent, trust, and digital literacy* (Figures 14, 15). Dynamic consent was described by stakeholders as the ability for individuals to control their data-sharing preferences in a flexible manner, similar to the concept of pausing data collection based on personal decisions:

*“So the dynamic consent is, like Donald Trump’s tax returns. He changes his income based on the way he feels that day. So it’s like today, I do not feel like giving consent, so I retrieve consent – we already do that […] You have the option to pause data collection. That is dynamic consent.”* (G1).

Dynamic consent was seen as especially important within Indigenous communities, emphasizing the importance of control over their own data for increased research participation and to address mistrust of science, research, and government – an inter-thematic connection made on the map. This approach was seen as a method to enhance compliance, participation, and empowerment, especially in communities with privacy concerns. Stakeholders also stressed the importance of trust as a key factor in addressing data security and privacy challenges. Stakeholders discussed how trust could be improved by ensuring secure systems, transparent compliance, and clear communication about privacy measures:

*“Answering, ensuring compliance. But there has to be a transparent system wherein the participants would be how we are ensuring their privacy.”* (G1)

In addition to dynamic consent and trust, stakeholders emphasized the need to address digital literacy challenges among communities to reduce hesitancy and enhance their comfort with data sharing, stating that:

*“So [there is a] need to increase the awareness of security how to store your data and how to avoid if they are setting up the digital [platforms]…”* (G1)

While mitigation strategies were not initially a part of the needs assessment, the findings reveal that it is a crucial aspect to investigate for the development of better digital infrastructures, with several key nodes of intervention: dynamic consent, trust, and digital literacy. The full list of subthemes and supporting quotes can be found in Appendix A.

## 4 Discussion

This rapid systems mapping study, which is a preliminary step in a long series of consultations planned for DiScO, utilized an innovative methodology of collating global stakeholder expertise and insights to inform the development and implementation of DiScO – digital observatory infrastructure to facilitate digital transformation of health systems within and across international jurisdictions via decentralization and democratization of technology. By decentralizing, this approach aims to avoid centralizing control, which often leads to reliance on external entities (58, 59). Instead, the goal is to empower communities and community leaders to provide control over the digital tools and processes they use to ensure that local stakeholders can directly benefit from and manage the technology, while tailoring it to their specific needs and contexts (22, 60). The purpose of the systems mapping exercise was to gather feedback from stakeholders who might use this infrastructure to engage with citizens in their own jurisdictions. Naturally, the basic infrastructure will need to be adapted to meet the specific needs of different communities and jurisdictions. This is a key decentralization aspect that DiScO can enable by engaging with citizens within each jurisdiction. The study design was informed by the need to incorporate systems thinking (17, 18, 61, 62) and systems integration (63, 64) to address existing gaps in global health systems in providing timely and effective healthcare (65). The key contributions of this study are: (1) engaging stakeholders at an international symposia, and in-person, which from a practical and strategic perspective is very important to minimize the logistical challenges; (2) In engaging the stakeholders, we have to prepare a year in advance by ensuring that our proposal was accepted by the international symposia, i.e., it underwent a peer-review; (3) the development of a complex systems map in real-time is very challenging and we were able to do this by applying the methods described in the manuscript; (4) We not only developed a systems map in real-time, but also corroborated it with qualitative data from the stakeholders to ensure that the approach is empirical; (5) Finally, the systems map is being used to inform the development and implementation of DiScO. The rapid systems map that was developed in real-time was expanded by thematically analyzing stakeholder discussions.

The primary purpose of DiScO is to coordinate decision-making with systems outside of health (i.e., food systems, social welfare) by monitoring, managing, and mitigating public health crises in the age of polycrisis (5). Leveraging ubiquitous devices for ethical surveillance, DiScO aims to implement rapidly adaptable, replicable, and scalable digital technology to address citizen-specific needs within and across jurisdictions. This decentralized approach to technology aims to break existing jurisdictional silos in addressing global crises by ethically leveraging big data from citizens, responding to rapidly evolving needs, and sharing evidence securely across jurisdictions to inform local solutions to global problems (5). This can be further enhanced through the incorporation of artificial intelligence tools that can analyze vast amounts of data in real-time, identify and visualize emerging trends, and provide actionable insights (66, 67). Not only can this approach improve the accuracy and timeliness of decision-making, but it can also enhance prioritization for jurisdictional decision-making to ensure that resources are allocated efficiently and effectively to areas of greatest need (68, 69). The implementation of the observatory will follow a co-creation approach by partnering with local stakeholders to ensure that the specific needs and contexts of each community are respected and integrated. The goal is to decentralize digital infrastructure so that each participating jurisdiction can adapt it to the needs of their citizens. This methodology ensures that community stakeholders have significant influence and power in the implementation process, fostering effective and sustainable health interventions tailored to local conditions. Given the complexity of this initiative, it is critical to incorporate diverse perspectives by engaging with key stakeholders from international jurisdictions (70, 71).

An established approach that has been shown to effectively capture diverse stakeholder input (72), particularly to capture systems thinking (73), is “systems mapping” (42, 74). A systems map can depict nuanced connections between factors across multiple levels and systems to visually illustrate the patterns and directions (i.e., relationships) of cause and influence on certain outcomes (41) – aspects that can inform the development and implementation of complex global infrastructure such as DiScO (5). While other studies have developed systems maps to inform public health research (41, 45), with one study conducting a rapid assessment of an existing systems map (75), no study to date has developed a rapid (i.e., real-time) systems map from start to finish, specifically by engaging with international researchers. To our knowledge, no rapid systems mapping exercise has been conducted thus far to engage global stakeholders to develop and implement digital infrastructure across international jurisdictions to monitor, mitigate, and manage complex public health crises. In particular, this is the first systems mapping exercise conducted in a global conference that enabled us to engage with international stakeholders in-person to generate a rapid (i.e., real-time) systems map to ensure cultural contexts were considered in its development. Within a collaborative setting, discussions unfolded between facilitators and stakeholders that informed the creation of a dynamic systems map in real-time. The purpose of incorporating this innovative design at global conference was to: (1) Ensure global stakeholder input through an iterative, yet rapid process, which is important to ensure digital health equity (76, 77); (2) Collect real-time data from stakeholders as discussions and capture the dynamism of the discussions in a systems map (75); (3) Validate the real-time data by transcribing and analyzing recorded discussions (25, 37).

This vision was presented to the stakeholders prior to initiating group discussions to foster a mindset aligned with systems thinking principles during the engagement. According to the WHO, there is significant potential to enhance the resilience and effectiveness of complex health systems using a systems thinking approach (78). Health systems, by their nature, are intricate networks of interconnected components, and research indicates that adopting a holistic lens through the application of systems thinking is beneficial in addressing health system challenges (14). The application of systems thinking has shown promising results across various public health domains. For instance, systems thinking helps to develop alternate prevention strategies, to better understand the causes of NCDs, and identify opportunities to improve health outcomes in the NCD domain (18). Moreover, significant contributions have been made to the planning and implementation of knowledge mobilization in public health policy and practice through the integration of a systems thinking approach (21). Initiatives guided by systems thinking often highlight the need to integrate health with other systems to achieve a more holistic approach to addressing complex health challenges.

In our rapid systems mapping study, beyond the expected connections that can be found in existing literature within a systems map, the rapid mapping exercise resulted in the identification of key nodes of intervention. This will inform will inform DiScO development and implementation by providing empirical evidence on how these challenges manifest in diverse contexts and how they can be addressed through a systems thinking approach. By engaging with decision-makers and researchers from diverse international jurisdictions, we ensured that cultural contexts were considered in the development of our systems map, which allowed us to capture how cultural determinants interplay with other factors to inform more culturally sensitive and effective health interventions.

A “node of intervention” is a key factor that not only has connections with multiple other factors, both within and across themes, but also has a significantly larger impact on connected factors within a systems map. These nodes of intervention go beyond simple diagrammatic summaries by elucidating potential action points for researchers, practitioners, and policymakers. Thus, addressing a node of intervention could have a ripple effect on multiple connected factors by showing how individual challenges are interrelated and how addressing one issue can impact others, which is important to both intervention design and the implementation of DiScO. The visualization between factors across multiple levels and systems through arrows and colors depicts a more comprehensive story and offers deeper insights to overcoming interrelated systemic issues. For instance, in terms of the ‘priorities’ identified by stakeholders, the three inter-connected nodes of intervention were *capacity building, co-creation, and equity.* This finding aligns with existing evidence which identifies the critical role of co-design and co-conceptualization in innovation digital health platforms (25, 27, 79) for enabling health equity (80, 81).

The systems map also highlights the key nodes of intervention to available ‘opportunities’ in enhancing *engagement* with citizens and patients, obtaining *community input*, and innovating rapid *knowledge translation and dissemination* approaches – aspects that are known to benefit from the incorporation of digital citizen science in health systems (24, 25, 53). These interventions can ultimately enable learning health systems, which continuously self-improve by systematically incorporating new data and feedback (82, 83). Learning health systems leverage digital tools to integrate patient data, clinical evidence, and community insights into a dynamic cycle of knowledge generation and application (82, 84–86). This approach not only enhances real-time decision-making (82, 87), but also ensures that health systems remain adaptive and responsive to the changing needs of the populations they serve to create more resilient, effective, and equitable health care environments that harness the full potential of digital citizen science (88–90) through strong partnerships and a shared vision of improvement across stakeholders (91). However, the map also highlighted interconnected nodes of intervention in terms of “challenges” (*digital/internet access, digital literacy, mistrust, and privacy and data security*) and “risks” (*cost–benefit and participant burden*). Internet inequity (24) and variations in digital literacy (92) are significant challenges (24, 92) that need to be acknowledged, and if possible, addressed to ensure the uptake of DiScO across global jurisdictions. For instance, there are wide variations of access to ubiquitous devices and data both within and across countries (93–95), thus, if not implemented thoughtfully, could exacerbate inequities. This systemic issue can hinder the implementation of DiScO if inclusive digital strategies are not adopted, which may include the integration of citizen- and community-driven approaches, tailoring technological implementation to local needs.

Privacy and data security are ongoing challenges in this digital age (96, 97). As technology continues to advance and the use of digital devices and online platforms becomes increasingly pervasive, the risks associated with data breaches, unauthorized access, and misuse of personal information are growing (98, 99). Consequently, maintaining robust data security measures and safeguarding individuals’ privacy rights have become critical priorities for organizations, governments, and individuals alike. Practical measures to address these confidentiality issues could include implementing end-to-end encryption, establishing strict access controls, and anonymizing sensitive data, while also developing clear data governance policies and creating transparent processes for individuals to manage their own data. A key mitigation aspect of DiScO is to decentralize technology (5) and enable data sovereignty of citizens to limit these risks by incorporating a global digital citizen science policy (24) that prioritizes citizen ownership of data. Decentralizing technology means shifting control away from centralized entities, such as large tech companies, and placing it into the hands of individual citizens and communities (58, 59). This empowers citizens to have greater control over their personal data, thereby enhancing trust and encouraging more active participation in digital health initiatives (22, 60). Similarly, digital citizen science approaches can increase the benefits to citizens and minimize potential participant burden by providing value in terms of real-time personalized support (6) – approaches that go beyond traditional and nominal incentivization of citizens to share data (32).

Four nodes of intervention emerged from analyzing partnership development: *industry partnerships, developers and computer scientists, citizens and communities, and organizations*. Industry partners across sectors are essential for advancing complex digital health initiatives (100, 101). For instance, developers and computer scientists, specifically with expertise in data storage, data management, and human-computer interaction are necessary in mitigating challenges and risks associated with obtaining big data, such as privacy and data security (102, 103). Similarly, engaging citizens and communities in data management can also mitigate challenges by aligning with digital citizen science for equal collaboration (24, 53). This could be done through the creation of advisory boards for community engagement, a participatory approach that aims to involve citizens in research processes (104, 105), as well as community-based organizations to facilitate authentic partnerships (106, 107).

The key nodes of intervention for ‘resources’ were: *funding, data sharing, and big data,* which identify three unique but intersecting aspects *–* consistent funding is needed to implement critical digital infrastructure to address public health crises (108), but if the funding is not equitable across global jurisdictions, then it is difficult to obtain equitable and representative big data (109, 110), which can have serious implications for development of inequitable artificial intelligence (111, 112). However, equitable data sharing can alleviate some of these resource challenges if digital citizen science approaches that DiScO aims to implement are consistently utilized to equitably transfer technology and obtain big data from low, middle, and high-income jurisdictions, irrespective of funding challenges (6).

The systems map also highlighted 3 nodes of intervention under ‘mitigation strategies’: *dynamic consent, trust, and digital literacy.* Dynamic consent and trust are linked to each other, where advanced human-computer interfaces can be used to ensure that citizens have control over consenting the big data they are sharing on an ongoing basis, i.e., digital citizen science informed technology that can improve trust (24). Digital literacy, which was also a key node of intervention within the ‘challenges’ theme is going to be a continuous issue across and within jurisdictions, which needs to address specific sociodemographic considerations (92, 113).

Finally, recognizing the interconnectedness between different thematic areas, the nodes of intervention that bridge these themes are arguably even more critical than those within each theme alone. For instance, key nodes of intervention were inter-thematically linked across priorities and resources. Specifically, discussions enumerated the importance of capacity building by effectively utilizing and sharing available big datasets across jurisdictions to optimize use of available resources (5, 6). Another inter-thematic key nodes of intervention link was found between challenges and mitigation strategies, namely mistrust and data sovereignty. Addressing mistrust through data sovereignty ensures that individuals have control over their data, which fosters a sense of ownership and trust in the data management process (24). A critical key nodes of intervention link was also found across the themes of partnerships and resources through organizations and international collaboration. Collaboration with organizations can maximize the exchange and adoption of best practices on a global scale if done through international organizations, such as the World Health Organization (114, 115).

For digital transformation of health systems, systems integration is imperative. Digital transformation of health systems refers to the integration of digital technologies into all aspects of healthcare delivery (116, 117) by reshaping the entire ecosystem of health systems to be more adaptive, data-driven, and patient-centered (116, 118). This process aims to create more efficient, accessible, and equitable health systems that can respond to modern challenges such as aging populations, rising non-communicable diseases, and global health disparities (42, 44, 45, 119). The push for digital transformation in health systems is driven by the need for real-time data collection, personalized care, and improved decision-making across healthcare services. This transformation can lead to better patient outcomes, more efficient use of resources, and greater engagement from citizens in their own healthcare journey (118, 120). One approach that can facilitate this is digital citizen science, which directly sources big data from citizens irrespective of their location – a social innovation approach for societal solutions (6). Increasingly, researchers are utilizing citizen science in interdisciplinary and intersectoral areas of research (53, 104, 121–125) to engage citizens in research efforts. Citizen science approaches seek to actively involve participants in all aspects of the research processes (35), from conceptualization and data collection, to knowledge translation and evaluation (35, 126). The rapid systems map enabled us to identify key factors that highlight a variety of strategies to enhance participant engagement, including the use of citizen science language for citizen empowerment, ensuring mutual benefit between citizens and researchers, and co-creation of initiatives with population interest in mind, which reiterate existing evidence (25, 35, 127).

### 4.1 DiScO governance and ethical implications

As a range of big data will be collected through DiScO, previously tested rigorous management protocols for data collection, access, protection, storage, security, and sharing should be implemented. All country-specific data will be securely stored in each region/country-specific database cloud servers. Country-specific research coordinators will be responsible for managing jurisdictional data collected to abide by country-specific legislative protocols for safe storage and transfer. All data will be encrypted, anonymized, and aggregated before storage, ensuring that jurisdiction-specific data is not personally identifiable. Additionally, data sovereignty is a key aspect of data management, particularly in decolonizing digital citizen science, thus citizens will have full control over their data access and sharing. Finally, to enable cross-jurisdictional learnings, data sharing will be key, which will advance real-time knowledge translation.

Leveraging ubiquitous devices, such as smartphones, for surveillance in health systems offers significant opportunities for real-time data collection and monitoring, while also raising several ethical considerations. Ensuring responsible use of this technology involves collecting personal health data with informed consent, secure storage, and strict access controls to ensure only authorized personnel (i.e., decision-makers) can view sensitive information. Participants must be fully informed about what data is being collected, how it will be used, and who will have access to it. Additionally, individuals should have the option to opt-in or opt-out of data collection without negative repercussions. Robust security protocols, including encryption and regular audits, are essential to protecting health data. Collected health data should be anonymized to prevent identification and the potential misuse of sensitive information, even in the event of a data breach. Efforts must also be made to ensure surveillance does not exclude or disadvantage certain populations to avoid bias in data collection and analysis.

Compliance with local, national, and international data protection and privacy regulations is critical for ethical surveillance using ubiquitous devices. Participants should have the ability to access, correct, and delete their data, and surveillance methods should respect their preferences and comfort levels. Continuous risk–benefit assessments are necessary to minimize harm and ensure surveillance aims to produce tangible health benefits. One method to overcome some of these ethical implications is digital citizen science, which ethically leverages big data from citizens. This involves providing participants with a clear “value perspective” for sharing their data, similar to how big technology companies incentivize citizens by offering valuable services or products in exchange for data sharing. In this way, citizens are not merely passive data providers, but active participants who understand the benefits of sharing their data (i.e., ethical data use with informed, mutually beneficial practices). By addressing these ethical implications, health systems can leverage ubiquitous devices for surveillance in a way that respects individuals’ rights and fosters trust in digital health initiatives.

While the rapid systems map was created in collaboration with physical activity and nutrition stakeholders, findings from this map hold relevance beyond these specific disciplines due to shared priorities across all fields of health research in obtaining ethical big data to transform health systems. For instance, data privacy is critical in all health-related fields due to the sensitive nature of the data collected (128, 129). Ensuring confidentiality and integrity of health data is critical not only for ethical reasons, but also to maintain public trust in research and healthcare systems – a link found in the systems map. Discussions with stakeholders determined that the implementation of data protection measures, including encryption protocols, secure storage systems, and access controls could mitigate the risk of leaked personally identifiable data. Similarly, wearable sensors and remote monitoring devices play a pivotal role in collecting continuous physiological data and contextual parameters to enhance the quality of care and patient outcomes (130). This physiological monitoring is applicable within emergency departments (130), clinical settings (131), and working environments (132). Thus, discussions around researcher priorities, opportunities, challenges, risks, partnerships, resources, and mitigation strategies that informed the development of the rapid systems map will be used to develop and implement DiScO to ensure potential benefits across disciplines and sectors.

### 4.2 Action plan for operationalizing DiScO

Based on the analysis of the final systems map, key methodological validations and intervention nodes were identified that will guide the development and implementation of DiScO. These steps, derived from the systems mapping exercise, are currently being implemented with Canada Foundation for Innovation funding:

1. Rapid systems methodology: This methodology, through a pre-post design, yielded key nodes of intervention, which need to be further explored by engaging a larger group of global stakeholders before implementation of DiScO.
2. Decentralization and democratization of technology: To ensure equitable digital transformation, decentralizing and democratizing access to technology across global jurisdictions must be an essential aspect to the deployment of DiScO.
3. Advancing digital literacy and internet equity: DiScO will aim to address and advance digital literacy and internet equity across populations, which are two key drivers for the success of the digital transformation of health systems.
4. Capacity building across stakeholder groups: Building the capacity of researchers, decision-makers, and citizens via DiScO across global jurisdictions is necessary for effective digital transformation of health systems.
5. Digital citizen science for systems integration: Digital citizen science will be central to catalyzing digital transformation of health systems using DiScO. This will enable systems integration via ethical big data surveillance.

An evaluation team is currently working on identifying relevant indicators for a process and outcome evaluation to ensure that DiScO’s implementation and impact are concretely measured. This may be conducted using digital ecological momentary assessments – short, user-triggered surveys administered via the observatory itself. This approach allows for real-time data collection, which will provide immediate insights into citizen and stakeholder experiences, and enable the observatory to adapt its approach based on user feedback.

### 4.3 Strengths and limitations

The primary strength of this study is its innovative design that ensured real-time data capture as well as validation of those data after qualitative analyses. Another strength is the logistical decision to conduct the systems mapping at a global summit, which facilitated the participation of health systems stakeholders representing eight countries. The expertise and input obtained through this approach would have been difficult to implement if rapid systems mapping were to be conducted as a separate event – difficulty in scheduling, extensive resources to bring them together, among other challenges were averted. While this study presents significant strengths and offers tangible action items for the development and implementation of DiScO, it also has some limitations. These include concerns related to sample size and representation, academic focus, geographic and economic limitations, and a limited exploration of the intersection between social and digital determinants of health. While a comprehensive systems map was developed during this study, this map must be replicated and validated by repeating this study with a larger and more diverse sample. Future efforts should potentially involve global events that bring together experts from a wider range of research fields, such as computer science, data science, digital health, epidemiology, and health geography, rather than predominantly focusing on physical activity and nutrition researchers. Another limitation is the potential bias introduced by the self-selection of participants attending a conference in Sweden. This group was not selected based on specific criteria to represent the full range of sectors or countries involved in this work. As a result, this group was predominantly composed of academics and researchers, which may not accurately represent the broader community or society. Furthermore, participants represented eight countries in total, with only one country, India, classified as a lower-middle-income country. This may have potentially skewed the findings as perspectives from high-income countries can overlook and not fully capture the unique challenges faced by health systems in lower-middle-income countries. Finally, although a broad range of determinants were discussed, this list is not exhaustive, particularly regarding the intersection of social and digital determinants of health – a critical aspect for the implementation and uptake of any digital initiative (133–135). Addressing these limitations in future studies may help to minimize potential biases in stakeholder participation, enhance data accuracy, and increase the generalizability of the findings across different contexts.

## 5 Conclusion

Rapid systems mapping at international symposia is a novel methodological approach to capture health system stakeholder expertise and input, particularly to understand complexity across international jurisdictions – an approach that can be replicated across disciplines and sectors to inform digital transformation of health systems. The development and implementation of DiScO, a platform for decentralization and democratization of technology, will take into consideration all the key nodes of intervention identified in the rapid systems map to ensure digital health for equity across global jurisdictions.

## Data Availability

The original contributions presented in the study are included in the article/Supplementary material, further inquiries can be directed to the corresponding author.
