# Supplementary material for: DiScO: novel rapid systems mapping to inform digital transformation of health systems
Source: Front Public Health. 2024 Oct 25;12:1441328. doi: 10.3389/fpubh.2024.1441328 (PMC11544543; doi:10.3389/fpubh.2024.1441328)
Supplement: Supplementary file 1 [file Data_Sheet_1.DOCX]

**Appendix A**

| **Theme**  ***Subtheme*** | **Supporting Quotes** |
| --- | --- |
| **1. Priorities** | |
| ***1.1 Capacity Building*** | *“Because we need to have a better collaboration between researchers and policymakers. – But communities as well and across different levels because we know that the needs and the priorities are the same at different levels of community level. There’s more collaboration and more, like, sharing resources and expertise.” (OGD)*  *“... particularly if we are working across – with partners across different countries, different jurisdictions, everyone has a different capacity, right. So if we have the tech capacities, someone might have – someone might have something else” (OGD)*  *“... maybe capacity building to allow for like-like across like multiple sectors to be able to intervene to make create intervention…” (G2)*  *“But building capacity – and because we live in this digital world – we can potentially exchange that capacity and share resources as well.” (OGD)* |
| ***1.2 Co-Creation*** | *“Co-creating physical activity programs with Indigenous youth in Northern Canada. We can also extend this point to say co-creating any such program with the community that you’re looking to serve in your respective jurisdiction, of course.” (OGD)*  *“That's where we have to work closely with the communities to have a response otherwise that could be really frustrating and perhaps even perpetuate your problem.” (G1)*  *“What digital citizen science is enabling us is to tap directly to the whole community at the same time, and that’s having quite an interesting effect on things like sampling bias.” (OGD)*  *“I think including adolescence, is – is our target group, but every kind of target group in the boat before asking the research questions and collecting data is important to know whether we are in the right direction or not.” (G1)* |
| ***1.3 Equity*** | *“You know, I've been asked this question, “why are you so focused on smartphones?” Because I don't care about smartphones. I was a late adopter of a smartphone. But what interests me is its market penetration, and it could be a tool of equity because everyone’s got it.” (OGD)*  *“And I mean, this is where I get a bit frustrated because if you talk about equity, people who don’t have enough money, people who don’t have enough time, flexibility with their jobs, where does physical activity actually rank on their priority? On their daily life? Right? We have to take that into context when we are building some of these things.” (OGD)*  *“Then you have the cool tech to make your apps gamified to take – we are sometimes taking the whole literacy out of the picture where – some kids have different literacy levels across a province or a country. So how do we make it more equitable? I'm not an educator, but can we create informed consent without words, without sentences, using a video? Animation?” (OGD)*  *“The interesting thing is lack of equitable access to Internet is an issue. When we go to smaller communities, we see kids grouping around libraries or McDonald's, which is an interesting intersection with nutrition altogether because they're getting free Wi-Fi, right?” (OGD)* |
| ***1.4 Value Perspective*** | *“So if I’m saying I’m this cool physical activity researcher, I’m taking this top down approach like “I want all your data and I’m going to write all these cool papers”. No one’s going to talk to me. When I go to these communities or hospitals, I actually don’t even use the term “research”. I talk solutions, right?” (OGD)*  *“Because Google is providing a value perspective. Google is giving me this cool app that I can use and navigate whether I’m in Sweden or Germany or Australia or South Africa. What are researchers doing? We're just taking the data and writing papers.” (OGD)*  *“Even if you show their own – tracking of their own data, that’s helpful for people to participate in their studies. Simple algorithm code you can write where there's a simple dashboard on their app and they are able to see what they have done that day. Google does it so intuitively, very easily.” (OGD)*  *“Now if you want to really capture environmental changes, you got to think not as – I think you can't think like a health researcher, you got to think like a marketing researcher and say, ‘How do I market this app? How do I create value perspectives where kids use it and take pictures of their environment and map?’” (OGD)* |
| ***1.5 Data Collection*** | *“... as a first priority I'd like to identify, improving of the surveys of physical activity, nutrition and to improve it – like making it continuous, making it [inaudible], improving validity, reliability, credibility within the survey.” (OGD)*  *“One is to collect survey – to collect data” (G1)*  *“All I do is research, but I’ll do better research if I don’t talk about research. How ironic is that? Because you’re talking about applied stuff, so if you’re going and saying, “Okay, we want to create a solution for you”, you get better data.” (OGD)* |
| ***1.6 NCDs*** | *“prevention of NCDs in urban slums and this is the area where that we work in in Pune. So the prevalence of NCDs is really, really high. And because these people are in transition and because they live in urban slums, there is always an issue with the exercise, with the food security, and even the quality of the food that these people eat.” (OGD)*  *“We have issues with obesity and issues with metabolic syndrome. We have type 2 diabetes in young people” (G1)* |
| ***1.7 Community Level Implementation*** | *“... how to integrate lived experience or citizen experiences? Not into surveillance, but into to drive solution building. You know, because if we're just looking at it from a high level” (G2)*  *“... you need to identify the local needs first before we design intervention definitely.” (G2)*  *“... improving surveillance of physical activity, nutrition, and for both overall priorities…” (G2)*  *“... so for priorities, improving surveillance of physical activity, nutrition …”*  *“... they know that physical activity helps, but that’s some kinds of physical activity that are not acceptable to communities. So, what kinds can we do that are A) acceptable and B) that are feasible given the environment that we're living in.” (G1)* |
| **2. Opportunities** | |
| ***2.1 Engagement*** | *“Because the majority of the populations that I work with are in what are known as reserves, so they live on reserve, which is communities that are further North in Canada … but everybody has a smartphone, so smartphones could be a way to create this.” (G1)*  *“Engagement of target participants and mutual benefit to both researcher and participants, absolutely.” (OGD)*  *“So, it's engagement of target participant and mutual benefit of both researcher and the participant.” (G2)*  *“... that is what the opportunity there in – That's the engagement of the stakeholder, the engagement of of the target population itself, India for us.” (G2)* |
| ***2.2 Knowledge Translation and Dissemination*** | *“Real-time translation and dissemination of knowledge, especially when you’re using something with a dashboard system which is able to collate statistics and present them graphically or visually. This is something that is a real advantage of being able to do KT in real-time.” (OGD)*  *“We often, in a traditional sort of research cycle, we wait till the end of the study to publish reports, maybe do a knowledge translation symposia or give something back to the communities whose data we collected. But using these platforms, we can do their knowledge translation in a much more savvy way and in a real-time way where they’re able to see their own data, whether it’s graphically or in different interesting ways of communicating that aggregate level data that gives them some idea of what’s happening in the community around them related to those issues. And even for decision makers or other stakeholders who are looking to actually implement some intervention or policy, they don’t have to wait six months, a year, two years until we give them the go-ahead saying, ‘We’ve analyzed the data, here you go.’” (OGD)*  *“Like, if you're targeting obesity and we conduct a question related to physical activity and diet and kind of disseminate findings in using graphs, colorful pictures, pie charts, all of that, it will be simpler. For them to understand their trajectories and the way that they are doing things and somewhere they could also kind of identify. I don't know mistakes or something which needs to be changed in their behaviours through the simple pie charts that we're projecting.” (G2)*  *“And sometimes it's not even like a visual. I think a lot of cultures might work better on storytelling, yes, or an oral kind of way of engagement.” (G2)* |
| ***2.3 Community Input*** | *“... I can see surveillance and evaluation of interventions or policies. That’s certainly one of the key reasons why people are using digital tools at all for research.” (OGD)*  *“If you want to do good surveillance, and if the digital literacy is low, it's a no brainer that we won’t be able to do it good surveillance, or ethical surveillance.” (OGD)*  *“... so the Community input I missed this so I get the surveillance and the evaluation the two sides of the intervention, right, the pre and the posting.” (G2)*  *“for evaluation of change of intervention and policy.” (G2)*  *“And but I think it can be used as well for evaluation of intervention new policies that are put into place when if we like create like a new intervention, there is a new policy that is created for improving access to good quality fresh products etc or to new urban design to improve physical activity in active transportation. If you put that into place we want to evaluate if it is working so we can work through like physical activity data, people working more, etcetera.” (G2)*  *“Or the other way could be identifying like key, youth – youth within each community and have each one with a smartphone for example, and they could then become the champions within their communities.” (G1)* |
| ***2.4 Screen Omics*** | *“... it is screen metabolomics. So the omics for metabolomics, except genomics and screen. So something related to how screen is playing a role in – I mean it's all pervasive sort of we have a lot of screen working – screen interaction.” (OGD)*  *“It's also about using screens to predict behaviors, such as how fast are you in typing with a keyboard. Yeah, and this can then predict, as it was said in our discussion, whether you will be in a car accident.” (OGD)*  *“There's a field actually called Screen Omix, that I have colleagues and that is their area of interest where they have developed ecosystems of data to capture everything that people are – even the pressure that people are putting when they're tapping away the spaces between, you know, it is unbelievable and there there's actually published results and, you know, huge big data sets showing how they can predict who's going to get into a car accident because of the sleep deprivation behaviours that are reflected in how they're using their smartphones. Or their computer screens, so it would seem like being able to harness that in a way that us, mere mortal people, who aren't digital, experts could could use them.” (G1)* |
| ***2.5 Marketing*** | *“you know how every time you're on your phone, you look at something, you get targeted adverts. If some genius within our world could do the same with, except creating an app that then would advertise like healthy behaviors. So, from time to time instead of getting an advert that comes and it's showing you some fancy clothing, if you're a child, a youth, who's maybe there is a periodic advert that shows you like something cool about being moving or something cool about some kind of positive health or mental health. You know, sort of like meditation is good so if we could sort of build these positive messages.” (G1)*  *“... we almost like want to create a counter narrative, because I'm always like, every time I get these targeted advertisements on my phone, I basically like scream in my head, I'm like ‘OK, why we don't the same with positive messages?’ If we could create messaging so that counters that, then for every message I get that says, ‘Is this you know? Could we have encounter by a positive message that says actually carrots are good?’” (G1)*  *“So, it can't be only a health-based app, we have to add something to it which makes it attractive to them” (G1)* |
| ***2.6 Data Mining*** | *“So you could do things like keyword searches is called natural language processing where if certain buzzwords keep coming up or trending, you're able to identify, maybe using a certain geography that in this geography these terms keep coming up, right? And that might be a clue to a certain issue or a certain hot topic that sort of thing, and like they're thinking they were thinking that maybe they could this to prevent like suicide attempts and suicide among the youth and people.” (G2)*  *“... but they're doing it in a less ethical way to sell you something, not to help you with anything?” (G2)*  *“... identify community or population … to identify right data, mining to identify population” (G2)*  *“Using Twitter data to identify groups where there might be high levels of mental health risk for youth that can should be like to identify the targeted intervention or target or at risk populations.” (G2)* |
| ***2.7 Empowerment*** | *“... he calls people instead of patients and participants and calls him citizen science scientists, it changes the whole perspective of their involvement. I wonder if that would work in in sort of the scenario you're describing that relationship would change through that power, balance as well. Wouldn't it? Yeah. Especially if they have some some voice in the the research questions themselves, like [...], I think was talking about. They get some benefit, but do they also get to to have input into their question generation or?” (G2)*  *“Yeah, they need to feel empowered to kind of answer those questions that we need answers to.” (G2)* |
| **3. Challenges** | |
| ***3.1 Competing Interests*** | *“There’s always competing interests. When multiple partners work together, everyone comes with their own agenda.” (OGD)*  *“... when we think about partnerships, we can easily brainstorm who needs to be at the table, but if we think about it, the reality of the work that we’re doing and who is part of our teams, we do tend to work in our circles, right? Because we work with like-minded people or with like-minded organizations that actually enable us to do the work the way we think we like to do it and it's very difficult to partner with some people maybe from industry or other places who don’t align in their philosophy or way of thinking or addressing a problem, and that’s where I think taking a high-level approach sometimes and looking at some things like a systems map or a larger framework allows you to see where and how you have that overlap.” (OGD)*  *“So for example, when we are looking to ask money from government or industry or elsewhere, it requires you having an appreciation and understanding of which angle to present your work, right? If you have an understanding of where you’re coming from, you can find that common ground to create that partnership. You don't have to align on everything, but that point of alignment is really critical to at least moving the needle forward with respect to one aspect of your priority area, right?” (OGD)*  *“The way the Indian Medical system works is there's a private hospitals and clinics and then the government hospital. And they don't get along with each other at all, so you know. [inaudible]. There is no collaborations between the two, so that actually is a very bad way to work.” (G1)* |
| ***3.2 Costs*** | *“One of the things we face is that none of our government grants supports behavioral change commits…” (G1)*  *“there are many grants which will fund you only for a year or two years and, so say, you are supposed to reapply at the end of the year. But it takes time, you know. There are three or four months between, if you’re lucky, or even more six months before the next funding comes in. So, what do you do with your participants? What do you do with your staff? And they’ll be cool, they say “you can hire new people.” But you know, especially when you’re working with participants in studies and not really with data. All our field staff have relationships with the participants, you know.” (G1)* |
| ***3.3 Digital/Internet Access*** | *“Yeah, access could be low, internet access could be low. It's huge. Without internet, nothing functions.” (OGD)*  *“Yeah, and Wi-Fi in particular. So, under-resourced communities. So, everybody doesn't – because we're working in remote parts of Alaska, and they have track phones that they don't have Wi-Fi. And so, they have to wait till the nutritionist, helicopters – and because there's no roads – so they helicopter in and then she can take their track devices back to her office, where there's Wi-Fi and then download it. So, you know, we can get Wi-Fi, as you were saying everywhere, that would be good.” (G1)*  *“...for example, in Canada, really, I think it's criminal [...] like I go to a community that's literally 300 kilometers from a major center, and there's zero connection zero. Like there's no phone network, there's like you're 300 kilometers away from a major center. Just to me, it is just because the people that live there, there is – they're not a priority to them…” (G1)*  *“So if you see in India, there are challenges for data collection in rural area through this digital Wi-Fi and yeah, there is yeah. Access to Wi-Fi and for some extent the elderly or the people who are, like, middle-aged, they don't use much of access to access to Wi-Fi with the low digital.” (G2)*  *“I talked about so low literacy and access and accessing that and everything is to preventing bias because some people will have more voice because they are more connected than other countries.” (G2)*  *“So increasing inequity and bias [are challenges] because if people have less access, they are really already like facing inequities and [...] because now that everything is getting digital, they're probably face even more restricted.” (G2)* |
| ***3.4 Digital Literacy*** | *“I see digital literacy, particularly in low SES populations. This is certainly a huge issue and a huge barrier because even if someone has a smartphone, it doesn't mean that they're able to engage with an app that we've developed or use it in a way that maybe is as useful as it could be to them, depending on their digital literacy level.” (OGD)*  *“we have actually worked on developing a digital literacy program for youth. [...] has been doing that because we found that in many of the communities that we work with, digital literacy is a barrier to participation, so we thought, “How do we address this?” So we've come up with a curriculum that could be administered by educators and schools or via an app depending on, you know, that still has yet to be designed, but could be adapted in that way to ensure that when someone is even reading the terms of service for our own digital platform, they confidently can go through those terms and understand what it is we’re seeing so they are more confident in knowing what they’re consenting to, what it is that we're looking to collect, and they're learning along the way how to navigate even other digital platforms so we know that that’s a risk in some ways, which we’ll get to of doing this work, but trying to mitigate that or supplement that by also providing digital literacy to the communities and the groups that we work with.” (OGD)*  *“...now illiteracy of course has gone down in the rule. So this is primarily the rural areas that I'm talking about urban area pretty, you know, sorted with respect to, you know they have access to Internet all of that but rural areas, there's still a little bit of illiteracy which goes on there so we kind of have to use mixed methods. That's right but even though there are some participants who are definitely illiterate, but they're smartness, that's absolutely amazing.” (G2)*  *“It's just about so they actually audio visual works how you communicate knowledge, mixed methods for health promotion” (G2)* |
| ***3.5 Mistrust*** | *“So the point I would add is variation of mistrust across jurisdictions. Yes, every jurisdiction has its own issues -- with mistrust that could be a big challenge.” (OGD)*  *“there is mistrust and when such a system is used in different jurisdictions, we have to take into consideration what the mistrust in that jurisdiction is related. That’s like, one size doesn’t fit all, right? Because we are trying to build standardization while taking into consideration variations in individual jurisdictions, the loss, the issues, cultural aspects and stuff like that.” (OGD)*  *“So the history of Indigenous communities is – there's a history of Western researchers stealing data or taking data not giving anything back, all sorts of things have happened in the past. So the idea of – the concept of sovereignty is very, very important [...] Because we're saying, ‘Who owns the data?’ and Indigenous communities would like to own their data, right? They value – and data sovereignty is big these days. Data is almost everything, right?” (OGD)*  *“...we got feedback that people were our participants. They were feeling observed all the time and many of them also had issues with data protection. Of course, we have ethical approval, and everything was well, I mean protected, but I feel that people still, especially in Germany, but I guess also in other communities, they do care about data protection, especially if we want to use WhatsApp for instance for collecting data. I think it is a big issue that people care about.” (G1)*  *“I just want to chime in here, like jurisdictionally speaking their different cultures and attitudes towards big data or apps, you're just talking about Germany and how much German roommate was paranoid that someone was gonna read this PowerPoint. So different countries are going in different directions. So, you see during code what happened in Taiwan and Israel and South Korea, where there's not a lot of legislation which is preventing the government from monitoring individuals. They were able to track people in the area of client. We could never do that in Canada. So, some of these things are not just the laws that determine it, but also, the cultures in those classes.” (G1)*  *“Like we have a more strict ethic committee or like more tenant because they are very old generation, not very into this. So you need a lot of lobbying with the policy to push it, even though it's like emergent, like world emergency for COVID. So it took took a lot of policy and navigating to really push through the study and for people, then it really requires some advertising like some public can pain to really also like.” (G1)*  *“And in Germany, the data security law and also people. Because the the like rude mentality to very against it is the major challenge that they don't want anyone to have any data an excess. They don't even use their real name on Facebook, they just they take privacy to another, level to and even for a study requirement during COVID time it was all rejected due to expect to work fine.” (G2)*  *“Yeah, like the even personally, like even everything according to GDPR, they still don't want to collaborate because they think it is not good to forget because it's a mistrust with the government issue and also researcher.” (G2)*  *“But the mistrust with respect to researchers is also prevalent in India…” (G2)*  *“The mistrust related to researchers and research projects in general and also system and powers. They are not really aware of what goes on, which is why they're skeptical, which is understandable but like a huge challenge in data.” (G2)*  *“The track tracing app data Tracing app is very low usage, even though it's completely anonymous decentralised, but because people don't understand what is decentralized and people just think no, there is Bluetooth opening then my data is not safe.” (G2)* |
| ***3.6 Validity and Reliability of Data*** | *“I want to know is that once these push notifications are sent out, how do you track if people work on them, like I send you a push notification that says drink water, but how do I know if after looking at that notification you've actually had a bit of water?” (G1)*  *“How do you track whether it has triggered the behaviour we wish to trigger? Because push notifications have been there for like about as long as I can remember, but how do I track if that push notification has really helped get you? Because then it becomes something like number screen – people always report numbers screened or numbers reached.” (G1)*  *“...we're getting more weight to the people who are more connected and there is one more reason that we may get wrong data because people fake most of the time, children, young, young children, they don't.” (G2)*  *“...they generally don't like answer right all the time they may give us the wrong. So like, what is this kind of like this? They may give us that is also getting the crappy data.” (G2)*  *“There is this variability, yes. And I think social desirability…people don't care about the, so they just pick everything in the center.” (G2)*  *“So to eliminate the cognitive decay, we essentially put those questions which are more important to us from an objective point of view in the beginning. So that the cognitive decay doesn't affect later partnerships. So I can say, let's do that and would it be a real community? Where are we with the time? Will be digital addictive like they are, are we?” (G2)* |
| ***3.7 Big Tech*** | *“But the problem is the political environment, right? All these big techs they will be funding all the politicians and the policymakers, and nobody listens to us” (G1)*  *“I live in Silicon Valley. These tech companies, a lot of them are social facings. They just need the right kind of push. Plus, it’s very competitive. So, if you get Google to share data. Facebook will then share data. Apple will then share. They're all within, literally, they're within 5 miles of where I live, and they care. They really do care, but you have to push them. They care a lot more than a lot of under other industries. I guess I should say you know, so we were saying, Fitbit, for instance, was great, and so Google took over Fitbit because Fitbit was sharing their data with all kinds of physical activity researchers. And then Google came in and all these legal things you know that made it really hard, but people are now pushing back on. And – I – they care, they really do care about their international, their global facing.” (G1)* |
| ***3.8 Lack of Resources*** | *“...for example in Canada, really, I think it's criminal like I go to a community that's literally 300 kilometers from a major center, and there's zero connection zero, like there's no phone network, there's like you're 300 kilometers away from a major center. Just to me, it is just because the people that live there, there is – they're not a priority to them…” (G1)*  *“So human resources, financial resources, I think that would be that's a major challenge.” (G1)*  *“So, the further away you go from a city center or a major metropolitan, the more difficult it is, I mean, all these problems are compounded with distance from the city center.” (G1)*  *“...nobody has the patience for this thing. It’s time consuming, it needs quality assurance…” (G1)* |
| ***3.9 Privacy and Data Security*** | *“...GPS, even that's that's considered, if any. Everything's anonymous is we. We have an app. Everything is anonymous. Everything's you never have any identification of the data. Except that the GPS. The geocoding is considered.” (G1)*  *“Geocoding has to be also decentralized […], otherwise they can use the geocoding with the timeline to analyze your work daily working pattern...” (G1)*  *“But still, even if it is anonymous. There is a GPS location of geotagging because if it could be a minority group, it could be children. It could be like a special interest group. There is maybe you can, so that would summarize.” (G1)*  *“This is data transfer and data storage meaning the data private, yeah. Because data transfer with data from the EU is a big deal.” (G1)*  *“It's all, you know, anonymized. It's encrypted, but we if, if anything is on someones phone, like the ID or whatever the address.” (G1)* |
| **4. Risks** | |
| ***4.1 Cost-Benefit*** | *“...leaking private data, government restrictions and laws, increasing inequity and creating bias, of course, addiction – that's an interesting one because this is something that comes up a lot when we’re even talking about physical activity [...] or mental health research where if we’re using a digital app to help improve youth mental health, but we know that smartphones are also especially used for social media [which] is contributing to poor mental health, where's the cost-benefit there, right?” (OGD)* |
| ***4.2 Participant Burden*** | *“Like when you always have to feel like answer, question, survey, etc., you’re overwhelmed by your phone. You get tired and you will just reply with less and less. ” (OGD)*  *“...we've been using something called ecological momentary assessments to make the process of completing what feels like a survey, but shouldn’t feel like a survey, a lot more expedient [...]. So that’s something that we also are actively working on as part of this to decrease the fatigue and increase the actual completion of some of this data collection.” (OGD)* |
| ***4.3 Risk to Science*** | *“One thing that drives me nuts is, I see it as a disconnect between the risks that are put on researchers having to have three different legal offices go through things versus people opening up Apple App and reading through blah blah blah blah blah blah blah hitting “I agree” and they're off to the races. Yet for us, they set this far that's so intolerably high that many of us can't even do the research. Or else it takes eight months, yeah, you know, I think there's a disconnect between the actual risks that science has created because we've got – we are very, you know, people sign human subjects consent form with all of the risks and the benefits. You know, Apple doesn't tell us the benefits of you know, I mean –. I think we need to address that, it’s getting out of hand, it seem to me around the world. So, I’m just going to make a note. Yeah, there’s a risk to science.” (G1)* |
| ***4.4 Data inaccuracy*** | *“So to eliminate the cognitive decay, we essentially put those questions which are more important to us from an objective point of view in the beginning. So that the cognitive decay doesn't affect later partnerships. So I can say, let's do that and would it be a real community? Where are we with the time? Will be digital addictive like they are, are we?” (G2)* |
| ***4.5 Excess Screentime/Digital Addiction*** | *“Too much screen time and too much digital addiction is one of the risk.” (G2)* |
| ***4.6 Increasing Inequity*** | *“So increasing inequity and bias because of people have less access, they are really already like facing inequities and because now that everything is getting digital, they're probably face even more restored.” (G2)* |
| **5. Partnerships** | |
| ***5.1 Industry Partnerships*** | *“The partnership with the industry emerged in our table as more important. Maybe because without including them in the boat, then we can just do research and then what is the change if they are not included?” (OGD)*  *“Industry definitely has to be a partner. Otherwise without them, we can fight what we want. Without their partnership, it's not happening.” (G1)* |
| ***5.2 Organizations*** | *“I think having the support of World Health Organization will help [inaudible].” (OGD)*  *“Community-based organizations, faith organizations…” (OGD)*  *“Whether they gather so it could be a school, it could be a community gathering center. It could be places of faith, places of faith.” (G1)*  *“Yeah, in other communities without faith, cooperation, nothing is moving. Some places if a faith group hasn't authorized it, it's not happening. It doesn't happen.” (G1)*  *“If you can get the imam of the mosque, yes, to support you, it really works with the community. They trust him, so they trust what he’s telling them. So, if we have him on our side or the village head on our side. Then the villages are more open to us coming in and talking to them, so that kind of partnerships, yes.” (G1)*  *“I have talked with global surveillance organization.” (G2)* |
| ***5.3 Developers and Computer Scientists*** | *“Human computer interaction scientists because we are [inaudible] and we don't feel very pleasurable looking at and it will impact on the usability and utility. So it’s really important to make the platform as easy and as pleasurable to use as possible.” (OGD)*  *“I would think partnerships with somebody who can develop the app for starters, because we don't have the wherewithal for it.” (G1)*  *“Yeah, I said partnerships with somebody who has the understanding of the app development, who can do what we are asking them because I can give you the idea, but I have no understanding of how that app is developed, what goes on in the background, and then once that app is developed to sustain supported, that would be something –.” (G1)*  *“And the data is stored safely, so we need somebody to help with that.” (G1)*  *“So, among researchers, so it could be researchers that are good in computer science or researchers who are in behaviour sciences and behavioural research.” (G1)*  *“So many partnership frame match easily care partnership for app development, for supporting app and storage, funding as always, and citizens.” (G1)*  *“...developer and human computer interaction.” (G2)* |
| ***5.4 Citizens and Communities*** | *“Yes, that's where we have to work very closely with the communities to determine what is done with that data, because that's definitely no longer under research jurisdiction and we need to protect the privacy of those people, but also ensure that they get adequate help. So, usually even those complaints or reports could be anonymized, and let's say the mayor of the community would at least be able to see the frequency of those incidents and where their company and someone could choose to disclose themselves, if they actually want to receive help from that decision maker. But this in some ways does require that at the back end, we're not just creating a new problem and having this solution. That's where we have to work closely with the communities to have a response otherwise that could be really frustrating and perhaps even perpetuate your problem.” (G1)*  *“So, our initial stage of any project is just developing or upon with the community that could be the village, that could be the school, it could be anywhere, it could be the urban slum. And only once we have an in with them, then yeah, we enter the community, yeah.” (G1)*  *“So I do for teaching. So we have a community of practice where basically regularly people meet and talk about challenges they face or they talk about what's worth if they've actually I become. Sometimes people might say this is the problem I'm having and then the community helps work workshop the the possible solution and then they might come back and tell you what's happened. So it's like it's usually around something, so I'm in a regular community based intervention teaching one and you could do a you could. So we can say it's a participatory do a citizen science one around activity also around, yeah, yeah.” (G2)*  *“It's usually for people who are the practitioners, so people doing. So in citizen science, I imagine it would be citizens and activists, as well as the various other partnership, one since you're getting a community centered participant LED research model, something like that.” (G2)*  *“They also called advisory councils where you have representation of different stakeholders. And in the case of a community of practice. It could be practitioners. It could be actual community members who come together to give that type of feedback about how to address something because they are representatives of their respective groups or respective disciplines, and they're able to share that in a more community advisory boards.” (G2)*  *“I guess easy to manage advisory Board without consulting the whole community. You called it community of practice. Consult with that community or that advisory.” (G2)*  *“... I always talk about this, like an authentic partnership between and amongst our researchers [and] policy markers at every level and the citizens themselves, so as equal partners, right, not just the talk not like the hierarchy, yes, as equals, so that everybody feels like they can be heard.” (G1)* |
| ***5.5 Diverse Partnerships*** | *“We'll consider that a euro Swedish sick resource, so this will be international collaborations.” (G2)*  *“I think it will going on International research collaborations. It has to be this has to be local so schools or CEO? Something at level as well.” (G2)*  *“Well, yeah. So I was thinking more like researchers, international collaborations and then political support and the diagram, yeah.” (G2)*  *“Yes, multi sector, right, it has to be a multi sector.” (G2)*  *“So it's so we've covered international partnerships and then local at local level as well. So and I don't know if it's the same a community of practice is that the same?” (G2)* |
| **6. Resources** | |
| ***6.1 Data Sharing*** | *“Some other interesting ones have come up here. Flexible data sharing, shared data platform – so public, secure data storage…” (OGD)*  *“Flexible data sharing, or at least not as strict.” (G1)*  *“Similar research all put their data into the shared platform and you can. I feel like with it, if it is not the easiest so that's good. Other than digital, almost is very and in India, basically, they encourage our government encourage the what we can say is international collaboration. To that very I think you use the word hyper scale. Yeah, yeah.” (G2)*  *“...I mean the idea that we would have a dashboard where everybody [could see data]. Yeah, local level data and then you could look – all the partners could [see] all the [data], you know, people on the network could look up walkability projects from communities around the world.” (G2)* |
| ***6.2 Funding*** | *“So some of the resources – money, money, money, no doubt. No doubt about that. Yeah. And that's something that I think once we start to making interconnections, that bubble would be bigger and bolder, because you can't really do anything without it.” (OGD)*  *“So, human resource, funding, technical know-how.” (G1)*  *“Talking about financial resources, I think. First of all, of course for doing research, we need more financial resources, but I think also, as an incentive for participants to join the research and also, we also need determination. From stakeholders, like we have funding schemes of three years, five years, seven years maximum, I think at least in Europe, however, especially when we want to take a life course research perspective, this takes decades. But is there enough determination from funding agencies, from governments, and especially when it comes to early career researchers, for instance, who have like 2-year contracts, 3-year contracts, while there are so many exciting things out there especially the digital sphere to do research about –. So yeah, that was the kind of determination I think is –.” (G1)*  *“They should put their money where their mouth is. Because every funding agent, Europe or in Canada, they are very good at, we promote researchers, especially educated researchers. But you never get the grant. Let’s you 3-4-5 times, writing this thing, and you don’t get it, like, how many times you have to write the same thing, you know, you’d think it’s a priority? “Yes, it is.” Okay, so give me the money to do it.” (G1)*  *“I think both your points are very good in addressing what OGD said. Many times, we have a long-term program and for us, like, they fund on year-to-year basis, and we are like, at the end of the year, where are we supposed to go? We can’t stop the research to wait until we get more money. We don’t have enough fundings to keep the research going, but that’s a huge problem.” (G1)*  *“Sustainable resourcing in a way.” (G1)*  *“That's terrible year to year. I mean, we had five-year grants but every year, we have to put in, you know, request, but we know it’s there and we can plan to hire people, and oh my goodness, that’s awful.” (G1)*  *“And that one of our experiences was that we were – we started six months of the study, was supposed to be a two-year study, then they said ‘No, we may pull out, we decided just to watch it for six months’.” (G1)*  *“Bridging funds I think are important.” (G1)*  *“Like so, we've never got any to start with. But essentially, like, there are many grants which will fund you only for a year or two years and, so say, you are supposed to reapply at the end of the year. But it takes time, you know. There are three or four months between, if you’re lucky, or even more six months before the next funding comes in. So, what do you do with your participants? What do you do with your staff? And they’ll be cool, they say “you can hire new people.” But you know, especially when you’re working with participants in studies and not really with data. All our field staff have relationships with the participants, you know. Because like, if you see Magda, who is our PhD student, despite the fact that, she worked in gestational weight gain, so despite the fact that, when we take the consent, we tell them, we cannot admit to you to hospital, we can’t do anything in this, you know. But once a woman goes into labor, she’s the one who gets the first call, you know. So, that’s how it is. That’s how it is. It’s a personal relationship which people build up. You can’t sack that person; it is not done. But funders don’t understand any of this. She’s given up.” (G1)*  *“So, I'm in fact, we've been looking for what is called core funding. So, ours is a fairly small institute. So, we're looking for some funds which will tide us over if there are, like, in COVID, you know. So, we have this charity clinic for children with diabetes and we are supporting 650 underprivileged type one children and they range from a few days old to now we've gone up to 22 because they're not employed by this. In COVID, we just lost all funding because all funding went to COVID. But what were we supposed to do with our children? So, in fact, that hospital supported all of that, yeah. Yeah, so everybody got insulins and glucometers and strips, and we lost only one child to go with, actually, and that too, was because she didn’t have a mobile phone. Otherwise, we would have reached that so – but the hospital picked it up, but you may not be so lucky every time. And this was like, you know, children’s lives at stake. So, the hospital guys said, you know, “we have a very good chairperson.” So, he said, “I don’t care, you just see, how we will deal with these kids.” But it will not always happen.” (G1)*  *“Always comes down to money, doesn't it?” (G1)*  *“Well, we need to put so what, yeah. More resources than just like the yeah, yeah, yeah. Great, great. The dollar signs resources.” (G2)*  *“It's been part think about human resources to manage the observatory. Think about the funding that would be needed. Think about the external resources as well. So funding is from the various sources. We can say different granting agencies. Plus in India we have this CSR which is corporate Social responsibility fund and so the government agencies they it is a lot of it, guy in the call the different calls different we can say different section in health and maybe I shift the IT and developer to resource rather than between nutrition, even the physical activity kind of thing so we can apply for that then.” (G2)* |
| ***6.3 Big Data*** | *“But what I mean by big data is – people like you, you can see the potential of what is big data? We have big data that people are not aware that they have.” (G1)*  *“So, you alone are generating big data everyday. Big data doesn't mean it comes from 100,000 people. Big data can come from one person themselves, because the velocity at which you’re generating data, because you own a smartphone or a device, is incredible.” (G1)*  *“Of course, but what I mean by big data champions is someone who has who can, who can have like good critical thinking to control source of data that can be used for creating, informing change in different areas because, of course, we are surrounded by data now, but I'm pretty sure that some of it is noise and some of it is – “ (G1)* |
| ***6.4 Data Storage*** | *“I’m thinking about data storage.” (G1)*  *“So, we need better data storage or platforms and space.” (G1)*  *“Data storage for sure. Like secure data storage.” (G1)* |
| ***6.5* Real-time Dashboard for Advocacy** | *“I think for advocacy, if you build a dashboard that tells you what's happening in real-time, that could be – I’m sorry to use this cliché term, but it’s a game changer, right? And yes, of course, you can aggregate data longitudinally temporally and create report cards more effectively. That solves a lot of issues in figuring out what's happening. It's not just data collection, but if you're able to push some interventions using ecological momentary interventions in real-time, you can potentially see changes as well and just, it might not work for 5 to 14 year old kids, but it could work for 14 to 18 if they have access to some devices. Or if it’s 5 to 14/13, whatever, I’m making up these numbers, you could potentially involve parent child diets and push the intervention to parents and this is doable.” (G1)* |
| **7. Mitigation Strategies** | |
| ***7.1 Dynamic consent*** | *“So the dynamic consent is, like Donald Trump’ s tax returns. He changes his income based on the way he feels that day. So it’s like today, I don’t feel like giving consent, so I retrieve consent – we already do that [...] You have the option to pause data collection. that is dynamic consent.” (G1)*  *“This is actually something we've had a back and forth with, even with our ethics boards. We work a lot with Indigenous communities and you just get into trouble and they might not necessarily want you to know where they're going or what they're up to, and that might actually prevent them from wanting to participate in your study at all, because they don't want you to have their data. But if you give them the option to pause that data collection or say, “I'll return when I feel like it.” Yes, I mean there's different challenges that we can talk about from a research perspective, but that actually improves their compliance and their adherence to the study in the long term, right, because we've given them those options.” (G1)*  *“It's the perception of dynamic consent that gets you better data.” (G1)*  *“I think this could be huge. This would be a huge node right there because it increases compliance, it increases participation, empowerment. You really think – if I give dynamic consent, it gives me confidence in being a citizen scientist, like I have control.” (G1)*  *“True, yes. And actually like this is a sidebar, but we have actually worked on developing a digital literacy program for youth. [...] has been doing that because we found that in many of the communities that we work with, digital literacy is a barrier to participation, so we thought, “How do we address this?” So we've come up with a curriculum that could be administered by educators and schools or via an app depending on, you know, that still has yet to be designed, but could be adapted in that way to ensure that when someone is even reading the terms of service for our own digital platform, they confidently can go through those terms and understand what it is we’re seeing so they are more confident in knowing what they’re consenting to, what it is that we're looking to collect, and they're learning along the way how to navigate even other digital platforms so we know that that’s a risk in some ways, which we’ll get to of doing this work, but trying to mitigate that or supplement that by also providing digital literacy to the communities and the groups that we work with.” (G1)*  *“There was a red one people actually read the term and agreement, they just click agree and then say because if you don't click you cannot use the app or the website. So it's like force and then what she's working on is to she's a lawyer, and then she's trying to build a service that actually make people actually look at like, highlighting or like a certain interface design.” (G1)*  *“So people actually know where to look at and what to look at or what? They're agreeing to and then also have to change dynamic consent.” (G1)* |
| ***7.2 Data Security*** | *“ensure secure data and all that. That should go into mitigation. And basically, data security is a challenge, right? It’s not easy.” (OGD)*  *“Yeah, I think the standard answer here is we always follow the most advanced encryption anonymization approaches, which evolves on sometimes day-to-day basis. That's what we want to do and that’s what we do. The other way – the other thing that we do is we host all the data in cloud servers which is outside of our organizational server ecosystem so it doesn't risk our organization. That helps with ethics because when you're talking about apps and digital platforms, particularly hospitals or institutions, they have to take care that there's no malicious attacks on their server system because someone else is using an application or a platform, right? So those two are critical. Cloud computing is huge for us, and that's what we try to do. The next level of, I would say, layering security is, except for phone numbers, we don't ask for any other information. And the phone numbers is only for multi-factor authentication so that we know that that person is trying to log in. Because you send a code to the same phone number and we all do multi-factor authentication. We try to log into a new system or something else. ‘Is this is this you logging in?’ They send us a code and they send us a text and then we enter the code and that's how we address it. So, I think we also have to take into consideration the laws that govern different jurisdictions. One of the things we’ve done is we look at the more stringent regulations like GDPR, which is in VU and say, ‘How can we improve it? How can we go beyond that?’ The interesting thing here is because we're not trying to sell the data, it’s easy for us to avoid those. How does – how do people make money out of apps based on data? Third party services. We don’t do that. So everyone's scratching their head and they're like, ‘How do you do this? Is this truly altruistic?’ It could be, but I think there is – there is a commercialization model in this while you do not sell the citizens’ data and I've had a conversation with a guy from Netherlands. His work – he's doing a startup on monetizing citizens’ data and he was reading my papers and we were having this conversation. I said yes, you can do it, but you will have to take the fight with some big tech guys. That’s because you are basically challenging their notion of making money by saying, ‘Our citizens can put a dollar value on their data’. I agree with that. That is an idealistic view of it. ‘You guys take my data and make a ton of money, why am I paying $80 a month for my data plan?’ So there's actual constructive ways of – we are doing some things that are truly constructive, but we're also pushing some boundaries, which are more academic and idealistic. I'm saying, ‘I want to put a price on my data. When I'm giving my data to you, I want a discount, I want to make money out of my own data’.” (OGD)* |
| ***7.3 Trust*** | *“You can do with the help of that mitigation that is trust…” (G1)*  *“Yes, like we say that we'll ensure that privacy.” (G1)*  *“Answering, ensuring compliance. But there has to be a transparent system wherein the participants would be how we're ensuring their privacy.” (G1)*  *“I think in the ethical approval always that you have to know where the cloud actually based and there are certain country you are not allowed to store your data there.” (G1)*  *Yeah, and and for example, a lot of our intervention use WhatsApp and chat bot and put a GDP as an example. So it will be done. No mitigation, yeah. But if you use one app or Facebook, it's part of meta then the cloud is not in your own country then that will become the problem. So always have to consider the cloud and one of the mitigation things that we do in some of our projects is there are.” (G1)*  *“Like then maybe you can check them out of the library to do the data collection or something, so it's not continual monitoring of, you know in the same way, but at least it's not.” (G1)*  *“Fine, give them a source. You well also may you know with we have a very simple app, but you know if someone has a really old phone. Or they haven't updated yet or something? You know, it creates all kinds of or or you know for ours. You have to do intentional data upload listening to them which is another way of maybe mitigating some of the risks and I wasn't sure if there was a you someone could collect the data on this side they don't want to upload it for, for, for us you have to do an intentional upload so it it's complicated, creates some barriers but yeah, but that for that we need a good infrastructure like kind of people behind working behind whole system, right, so yeah.” (G1)* |
| ***7.4 Digital Literacy*** | *“So maybe it's a literacy that you can improve it. Maybe it's a motivation you can improve.*  *It sounds like they're very digitally illiterate. They're just hold the privacy set up.” (G1)*  *“Yeah, so very smart is. So it will be likely to receive like education. Like increasing literacy, increasing literacy, digital literacy. Understanding and understanding reasons for hesitancy, even that. Sounds like advocacy to the decision maker for the policy and law change, but also going the other way is working with the Community citizens.” (G1)*  *“Maybe even that we call them like those different groups that represent the communities and the different.” (G1)*  *“Yeah, happens them to then ask them what would maybe persuade them to be more comfortable sharing the data.” (G1)*  *“So [there is a] need to increase the awareness of security how to store your data and how to avoid if they're setting up the digital [platforms]...” (G1)*  *“Let me yeah, to increase how do you like do you need a different mitigation strategy to some of the things we said, like increasing literacy in literacy in communities that we spoke so I would say like target specifically this population and also improving their education like all the targeted with improving the access to the device and capacity building also is required, otherwise there are we'll focus for deployed in the villages, they are having them on app on their mobile phones and they are collecting data.” (G1)*  *“House to house survey they are doing and they're liking data nowadays. The lady who is not much well educated they are also doing that. So I think it is slowly improving, but still we need to definitely. We also have intergenerational projects where, you know, if you have a kid that's going with an older adults.” (G1)* |
| ***7.5 Global Resources*** | *“Just maybe like extend with the access to the resource community global issue being the mitigation.” (OGD)*  *“Because you have no digital tech access. I think it can be so low, they may be extending the access to be in the mitigation.” (OGD)* |
| ***7.6 Behaviour Change Communication Model*** | *“Feel we can't really go on only about rules and regulations by the government or some kind of some sort of governing authority, because at the end of the day, it all comes to a participant oriented research that we're looking at so it has to be, so I think something like a behaviour change communication model.” (G1)*  *“Of course it would take a little more time than our usual model methods, but I think it is the more sustainable way of kinding of kind of, you know, disseminating or spreading knowledge in general of what people are hesitant about. I mean, they're not really, they're really hesitant of not sharing any information, so there's kind of I won't say paranoia which will be a little but somewhere on lines of those insecurity and things progress in security. And also the colleagues work they're trying to use the behaviour model to understand the people's decision on the consent making.” (G1)*  *“Is it lack of capacity like knowledge or lack of motivation of like they don't think they are part of the community? Or contributing to something or is it?” (G1)*  *“We can see that we will, we will change communication.” (G1)* |
